# A proposed magnetic resonance imaging grading system for the spectrum of central neonatal parasagittal hypoxic–ischaemic brain injury

**DOI:** 10.1186/s13244-021-01139-7

**Published:** 2022-01-24

**Authors:** Shalendra Kumar Misser, Jan Willem Lotz, Stefan-Dan Zaharie, Nobuhle Mchunu, Moherndran Archary, Anthony James Barkovich

**Affiliations:** 1 Lake Smit and Partners Inc., Durban, South Africa; 2 Department of Radiology, University of Kwa-Zulu Natal, Faculty of Health Sciences, Nelson R Mandela School of Medicine, Durban, South Africa; 3 Department of Radiodiagnosis, Faculty of Medicine and Health Sciences, Stellenbosch University, Cape Town, South Africa; 4 Department of Anatomical Pathology, Faculty of Medicine and Health Sciences, Stellenbosch University, Cape Town, South Africa; 5 Biostatistics Unit, South African Medical Research Council, Durban, South Africa; 6 School of Mathematics, Statistics and Computer Sciences Centre for the AIDS Programme of Research in South Africa (CAPRISA), University of KwaZulu-Natal, Durban, South Africa; 7 Department of Pediatrics, University of Kwa-Zulu Natal, Faculty of Health Sciences, Nelson R Mandela School of Medicine, Durban, South Africa; 8 School of Medicine, University of California, San Francisco, CA USA

**Keywords:** Hypoxia–ischaemia in term neonates, Brain, Magnetic resonance imaging, Massive paramedian injury

## Abstract

**Aim:**

To describe the spectrum of parasagittal injury on MRI studies performed on children following severe perinatal term hypoxia–ischaemia, using a novel MRI grading system, and propose a new central pattern correlated with neuropathologic features.

**Methods:**

MR scans of 297 patients with perinatal term hypoxia–ischaemia were evaluated for typical patterns of brain injury. A total of 83 patients that demonstrated the central/basal ganglia–thalamus and perirolandic pattern of injury were categorised according to the degree of severity. The perirolandic injury was graded by the degree of interhemispheric widening, paracentral lobule involvement and perirolandic cortex destruction leading to a tiered categorisation. Of these 83 patients, 19 had the most severe subtype of injury. A detailed analysis of the clinical data of a subset of 11 of these 19 patients was conducted.

**Results:**

We demonstrated the mild subtype in 21/83(25%), the moderate subtype in 22/83(27%) and the severe subtype in 21/83(25%). A fourth pattern was identified in 19/83(23%) patients with a diamond-shaped expansion of the interhemispheric fissure, concomitant thalamic, putaminal, hippocampal and other smaller substrate involvement indicative of the most destructive subtype.

**Conclusions:**

We propose a new MR grading system of injury at the parasagittal perirolandic region related to severe, sustained central perinatal term hypoxia–ischaemia. We also introduce a previously undescribed pattern of injury, the most severe form of this spectrum, seen especially after prolongation of the second stage of labour. This constellation of high metabolic substrate, targeted tissue destruction is consistently demonstrated by MRI, termed the *massive paramedian injury* pattern.

**Supplementary Information:**

The online version contains supplementary material available at 10.1186/s13244-021-01139-7.

## Key points

- Parasagittal injuries are largely presumed to be due to partial prolonged hypoxia–ischaemia.
- Central patterns of hypoxic–ischaemic injury may also result in parasagittal cerebral destruction.
- A gradation of central brain injuries following severe, sustained hypoxia–ischaemia is showcased.
- We describe this spectrum of central type hypoxic–ischaemic parasagittal brain injuries.
- The *massive paramedian injury* subtype entails complete destruction of the central motor brain core.

## Background

The pattern of hypoxic–ischaemic brain injury (HIBI) in cases of profound ischaemia has been well described [1–6]. There is generally a variable degree of involvement of the basal ganglia (especially the dorsal putamen) and the ventral thalamus which combines to create the basal ganglia–thalamus (BGT) pattern [2]. Associated perirolandic injury, which may be of varying degrees of severity, is found in combination with the central nuclei destruction [2, 4–6], which we refer to in this review as the Rolandic-basal ganglia–thalamus (RBGT) pattern.

The term parasagittal cerebral hypoxic–ischaemic brain injury has been attributed predominantly to watershed territory involvement in partial prolonged type of HIBI [7]. The parasagittal cortex on either side of the inter-hemispheric fissure is traditionally recognised as a watershed zone, located between the major arterial territories supplying the cerebral cortex. There are however instances where a perirolandic injury may extend to the far reaches of the primary motor cortex or Brodmann area 4 (BA4) and into the premotor cortex or Brodmann area 6 (BA6), including the supplementary motor area (SMA) and association cortex. It is not uncommon for radiologists to report these injuries as “mixed patterns” of injury (with suggestion of partial watershed zone involvement in the parasagittal cortex owing to the exaggerated boundaries of the area of encephalomalacia). However, careful examination reveals continuity of the lesions with no intervening normal cerebral substrate, indicating that all the affected structures are contiguously destroyed, most likely due to a common pathophysiological mechanism [8].

Contrary to the appearance in monkeys, the area occupied by the agranular area BA6 is much larger than the adjacent gigantopyramidal area BA4 in humans [4], despite the fact that BA4 and BA6 are cytoarchitecturally very similar (both unusually thick, with lack of an internal granular layer and indistinct external granular layer). Neural networks connect these cortical areas with the basal ganglia as well as the thalamus and primary visual cortex, all highly metabolically active areas of the brain in a term neonate, and all demonstrating high N-methyl-D-aspartate (NMDA) receptor expression [9, 10]. It is, therefore, highly likely that near-complete injuries to these areas (as in the massive paramedian injury subtype) would be seen in an exaggerated form of profound ischaemia which is severe but also sustained for a more prolonged period of time [8].

## Aims

The purpose of this study is to describe the spectrum of parasagittal brain injuries identified at the perirolandic region in term neonates attributable to severe central type hypoxia–ischaemia. Selected examples are used to show the extent of injury in each grade and the associated structural damage incurred, remote from the parasagittal area, including the basal ganglia, thalamus and other important substrates. We intend to show that in addition to parasagittal injuries due to partial prolonged ischaemia, there is a gradation of injuries also identified at the parasagittal cortex, attributable to profound and sustained hypoxia–ischaemia of the central injury subtype.

## Methods

A comprehensive database of 297 children with clinically suspected term neonatal hypoxic–ischaemic brain injury was enrolled into the study. The patients were referred to the imaging centre for the evaluation of brain injuries related to hypoxia–ischaemia and this sample population compares well with prior studies [5, 6] and is considered representative of the spectrum of HIBI in term neonates. Imaging was performed at different times after the perinatal injury, dependent on clinician referral, and showed a range of patterns of injury in the chronic phase of HIBI. The MRI scans were all performed on 1.5 T Siemens Scanners. Sagittal volumetric, 1 mm slices GE 1900/2.95 (TR/TE (msec)) and coronal volumetric Inversion Recovery (IR) 1.1-mm slices SE 4000/363 (TR/TE (msec)), coronal IR through temporal lobes, axial T2, axial FLAIR, axial diffusion-weighted/ADC, axial susceptibility-weighted and coronal T2-weighted sequences were obtained in all patients. The MRI studies of these full-term infants were retrospectively independently reviewed by the principal investigator (S.K.M.) and co-investigator (J.L.) with neuroradiological expertise of 15 years and 30 years, respectively. From this database of 297 patients, we classified injuries into the four major patterns of HIBI [8] as per classification in Table 1.

**Table 1:** The four main MRI patterns in patients with hypoxic–ischaemic brain injury [8]

Subtype of HIBI	Anatomical structure involved	Type of insult
Central RBGT pattern	Deep Nuclei/Perirolandic cortex/Hippocampus	Profound hypoxic episode
Partial Prolonged or Watershed Pattern	Cerebral Intervascular Watershed Areas	Prolonged, moderate or intermittent
Mixed RBGT + Watershed Pattern	Deep Nuclei/Cortex/Watershed Areas	Severe, variable in duration
Cystic Encephalomalacia	Cerebral Cortex White Matter/Basal Nuclei	Total Anoxia

*RBGT* Rolandic basal ganglia–thalamus pattern, *Deep nuclei* thalamus, putamen

The studies were evaluated for morphology and signal abnormalities of multiple specific structures in the brain using a devised grading system. A simple 0–3 scoring system was applied to each region independently including 0 = no injury, 1 = mild injury (less than a third of the structure was injured), 2 = moderate injury (more than one third, but less than two thirds of the structure was injured) and 3 = severe injury (more than two thirds, with up to near complete destruction of the substrate). Particular attention was given to the substrates previously enumerated as being high metabolic areas of the brain including the perirolandic sensorimotor cortex, paracentral lobule, deep grey matter nuclei (especially putamina and thalami), as well as the brainstem, the hippocampi, the superficial and deep white matter, the visual cortex, the primary auditory cortex, insular, mammillary bodies, fornix and the corpus callosum [8]. Each study was assessed with a view to accurate grading of the injuries at each specific substrate. Sixteen separate substrate assessments, listed in Table 2, were initially undertaken for each patient. These areas of involvement were individually graded by each investigator and there was a high degree of inter-observer agreement with a weighted kappa of > 0.8. Regarding the basal ganglia, evaluation of injuries focussed upon the posterior aspect of the putamina and at the thalami, predominantly the ventral lateral aspect. Hippocampal injuries were documented based upon assessment in the coronal plane with relevant inversion recovery sequences planned according to the triplanar axes of the hippocampus. We subsequently focussed the evaluation to 5 specific parasagittal features listed in bold below (each evaluated from 0 to 3 as above) leading to a potential maximum parasagittal score out of 15. In reviewing the perirolandic injuries, careful note was made of the involvement of the paracentral lobule (PCL) including the supplementary motor areas, the superior frontal gyrus, precentral gyrus, the postcentral gyrus and the degree of interhemispheric fissure (IHF) widening.

**Table 2:** Key features of each subtype of parasagittal central hypoxic–ischaemic injury

	Mild	Moderate	Severe	Massive paramedian
Parasagittal structures affected	Sensorimotor cortex	Sensorimotor cortex, SMA	Sensorimotor cortex, SMA and more of the PCL	Sensorimotor cortex, SMA, PCL, extending to the SFG and SPL
Boundaries of the injury	Limited to perirolandic cortex only	Perirolandic cortex and anterior part of the PCL	Perirolandic cortex and PCL	Laterally to edge of central sulcus, anteriorly and posteriorly to the edges of the IHF
White matter destruction	Nil	Some reduction in WM volume	Slightly more WM volume reduction	Severe decrease in overall WM volume
Interhemispheric fissure widening	Apposed paramedian gyri	Slight widening of the fissure at the PCL	Inverted V-shaped opening of the fissure	Diamond-shaped widening of the fissure
Central cortico-spinal tract destruction	Usually none or minimal along the long tract	Mild hyperintensity along the long tracts	More significant destruction of the central WM tracts	Near complete destruction
Insular Cortex	Spared	Minimally involved	Moderately involved	Near complete destruction
Mammillary Bodies	Usually spared	Mildly injured	Moderately injured	Severely atrophied
Heschl Gyrus	Usually spared	Mildly injured	Moderately injured	Severely injured
Hippocampi	May be slightly decreased in volume	Moderately decreased in volume	Severely decreased in volume	Complete destruction
Calcarine cortex	Usually, normal	Mild signal change	Moderate signal change	Significant signal change and atrophy
Corpus callosum	Mild central CC atrophy	Moderate central CC atrophy	Marked central CC atrophy	Near complete central CC atrophy
BGT involvement	Mild signal change	Moderate signal change up to less than 50% volume involved	Marked signal change greater than 50% volume involved	Spongiotic cavitation at putamen and thalami

*PCL* paracentral lobule, *CC* corpus callosum, *SMA* supplementary motor area, *SFG* superior frontal gyrus, *SPL* superior parietal lobule, *IHF* Interhemispheric fissure, *WM* white matter

### Statistical analysis

Categorical variables of the key features of each subtype of parasagittal central hypoxic–ischaemic injury were expressed as frequencies and percentages and compared by using either the Chi-square test or, if there were less than five observations in any subgroup, the Fisher exact test. The logistic regression was used to assess factors associated with massive parasagittal central hypoxic–ischaemic injury versus mild, moderate and severe subtypes combined. A two-tailed value of *p* < 0.05 was considered to indicate statistical significance. All statistical analyses were conducted by using Statistical Analysis Software (SAS), version 9.4 (SAS Institute Inc., Cary, NC, USA).

## Results

**Fig. 1:**
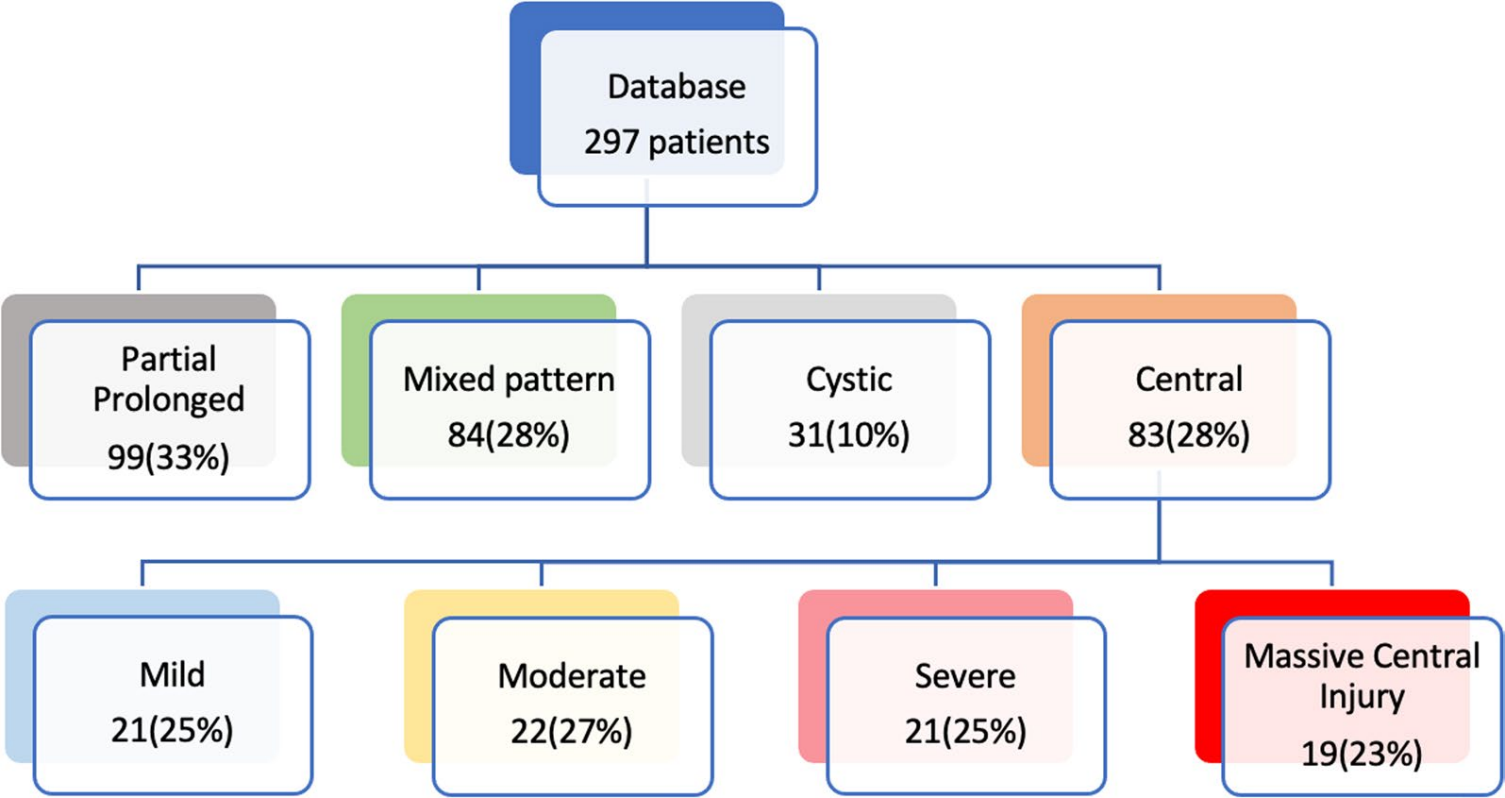
Schematic diagram of the sequence of derivation of the individual subtypes from the sampled database

The partial prolonged or watershed pattern of injury was seen in 99 (33%) patients, multicystic encephalopathy in 31 (10%) and a mixed pattern of injury with combined RBGT and watershed injuries in 84 (28%). There were 83 cases of true central (RBGT group) pattern of injury. The derivation of the individual subtypes of central HIBI from the complete database of 297 patients is shown in Fig. 1. All 83 patients with the RBGT pattern had some signal abnormalities associated with variable degrees of cortex atrophy around the central sulci, probably because the perirolandic cortex is the most mature at this age and, therefore, is the most metabolically active having the greatest need for energy substrates. The involvement of the perirolandic cortex was highly variable. In the mild subtype, the injury was sometimes limited to the precentral gyrus or post-central gyrus alone without involvement of the other lip of the sensorimotor strip often slightly asymmetrically. With increasing severity of injury, there was a proportional increase in the perirolandic signal change, again, probably related to the higher metabolic requirements. The degree of associated parasagittal atrophy was also noted to progress in tandem with the severity of insult and hypoxic–ischaemic brain injury in other remote substrates. In the moderate subtype of parasagittal injury, there was separation of the cortex at the perirolandic region, with slight separation of the interhemispheric fissure; not surprisingly, this atrophy was markedly increased in the severe subtype with widening of the sulci (severe as per global cortical atrophy scale) [11] and the interhemispheric fissure.

Concerning the basal ganglia, a gradual progressive involvement of the putamina was seen extending from the most posterior aspect to the central and ventral aspect with increasing severity of hypoxic–ischaemic injury. Similarly, the thalamic injury was noted to be isolated to the ventral lateral nucleus in the milder subtype, with progressive extension to involve a greater area and more of the central nuclei in the higher grades of injury. Again, this results from the fact that less severe reduction in energy substrate causes injury mostly affecting areas with higher metabolic demands (usually the most mature areas); as the reduction in energy substrate becomes more severe, more areas are involved. Progressive destruction was also noted of the insular cortex, Heschl gyrus and visual cortex from the mild to more severe subtypes, congruent to the perirolandic, putaminal and thalamic injuries. Variable deafferentation thinning of the corpus callosum, especially of the body and isthmus, was noted correlative to the perirolandic sensorimotor cortex destruction.

**Fig. 2:**
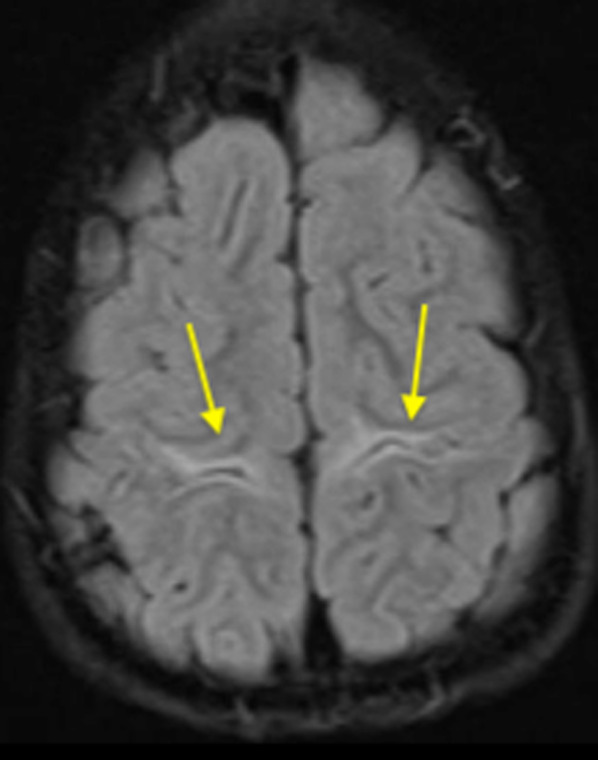
The mild subtype of perirolandic (yellow arrows) primary motor cortex and postcentral gyrus involvement

In the mild perirolandic (Grade 1) subtype, (*N* = 21/83, 25%) the central sulcus is outlined by hyperintensity on the axial FLAIR sequence, optimal to show (as in Fig. 2) this often-subtle injury. In some children, the injury may be limited in size and located in the pre-rolandic or post-rolandic lip of the sensorimotor cortex, with sparing of the rest of the cerebral mantle. When motor cortex injury predominantly involves the “hand knob” region of the homunculus, bilateral upper limb function is affected and there is no appreciable widening of the interhemispheric fissure. The parasagittal score in this subgroup did not exceed 6/15 and the average score was 3/15.

**Fig. 3:**
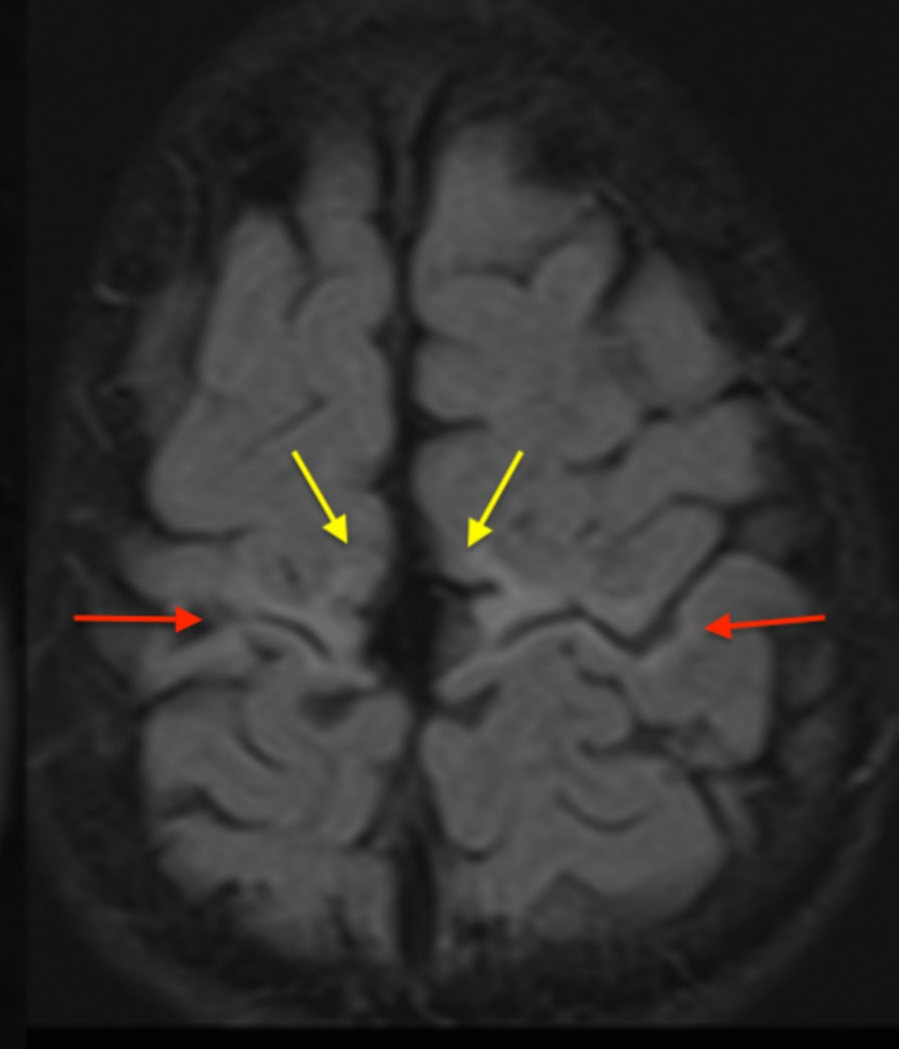
The moderate subtype of perirolandic injury (red arrows) including partial SMA involvement at the ventral aspect of the PCL (yellow arrows)

The moderate perirolandic (Grade 2) subtype (*N* = 22/83, 27%) is manifested as partial injury of the ventral aspect of the paracentral lobule in addition to the perirolandic injury. A particular involvement of the SMA is manifested in this subgroup (shown in Fig. 3) resulting in a slight widening of the interhemispheric fissure, in the region of the paracentral lobule anteriorly with sparing of the margins of the paracentral lobule, the lateral margins of the central sulci and absence of extension towards the superior frontal gyrus (SFG) and superior parietal lobules (SPL). The average parasagittal score for this subgroup was 5/15.

**Fig. 4:**
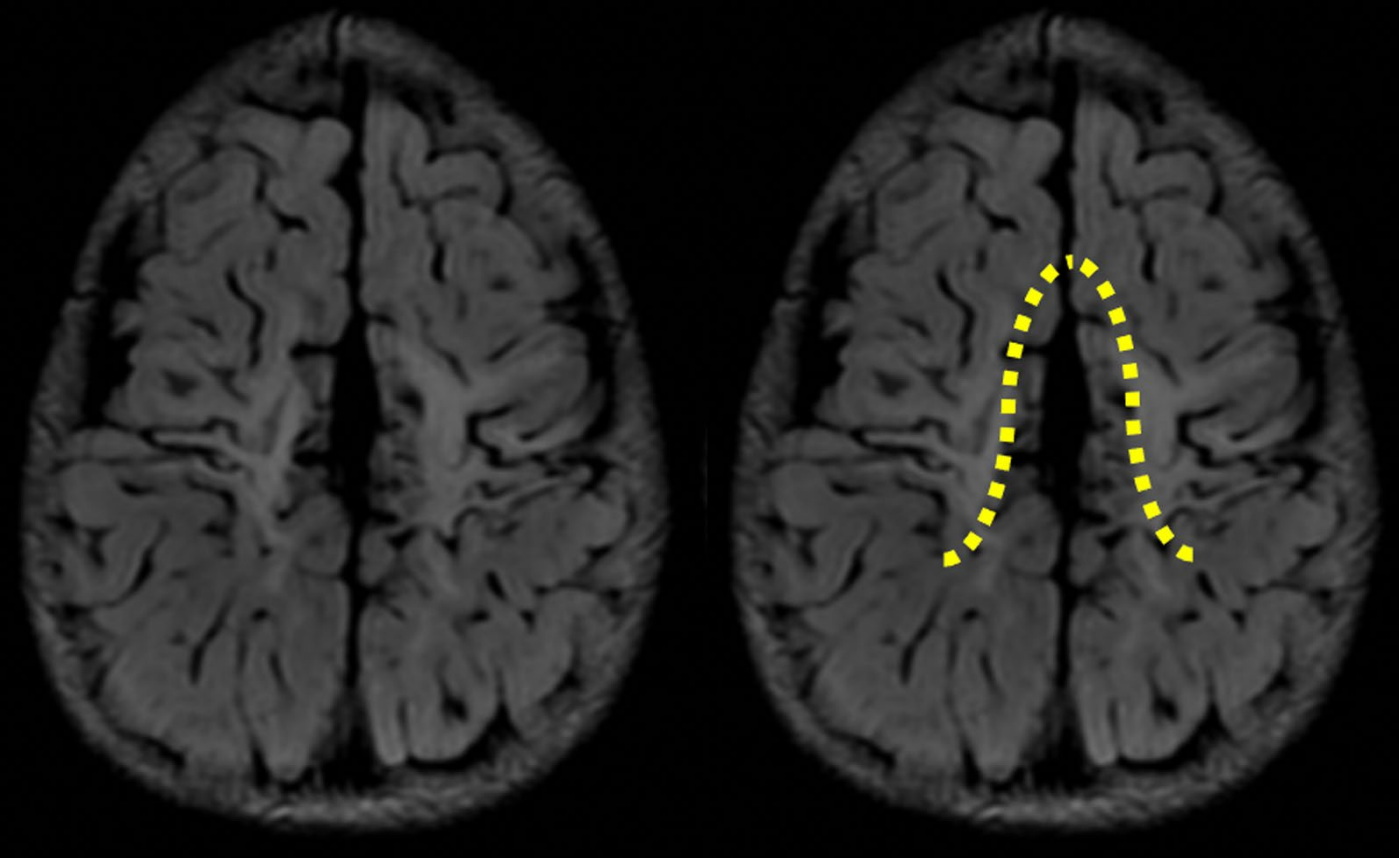
The severe subtype of perirolandic injury with greater PCL involvement and marked localised atrophy, widening the interhemispheric fissure, with an inverted-V configuration (yellow dotted line)

The severe perirolandic (Grade 3) subtype (*N* = 21/83, 25%) is associated with greater destruction of the anterior paracentral lobule including more of the SMA and surrounding premotor association cortex, more marked atrophy of the pre-central and post-central gyri, and greater widening of the interhemispheric fissure which has an inverted-V configuration, open dorsally and with a closed apex anteriorly at the superior frontal gyrus level (Fig. 4). The average parasagittal score in this subtype was 10/15.

**Fig. 5:**
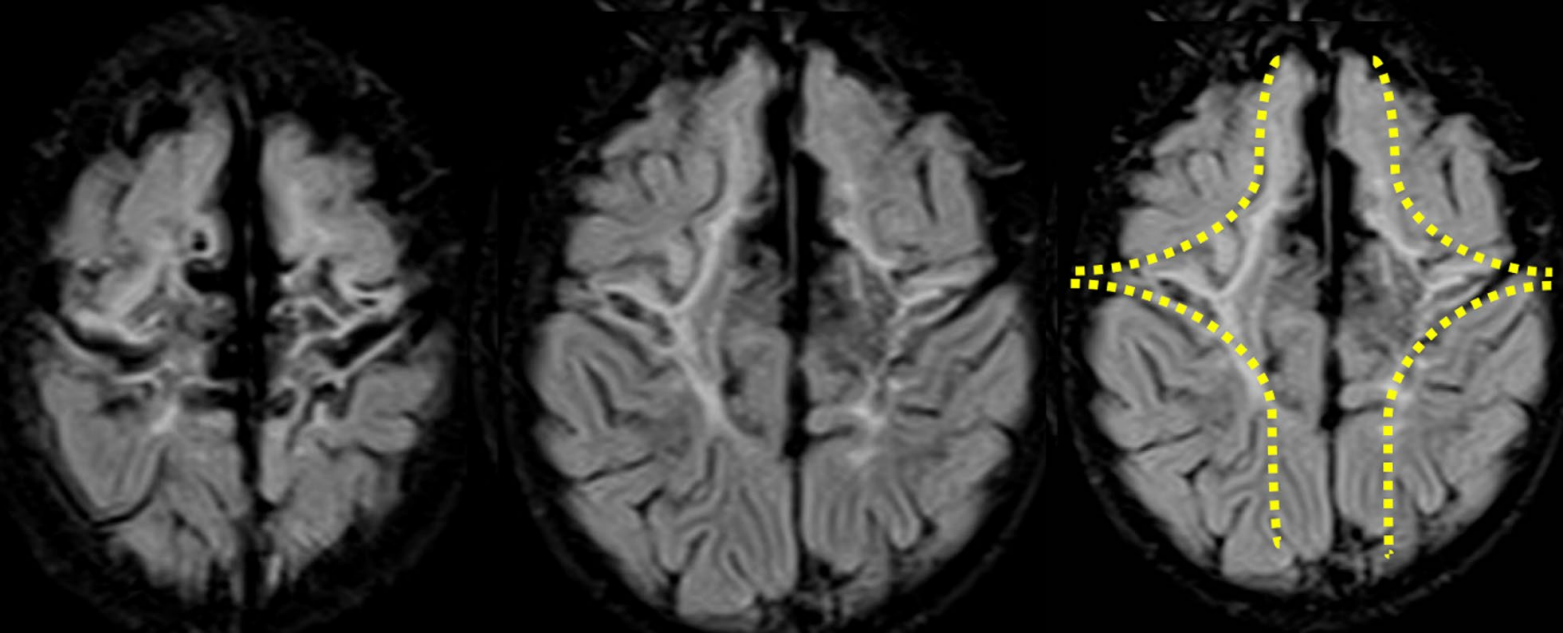
The MPI subtype of parasagittal cortex injury including complete destruction of the SMA, with secondary diamond-shaped (yellow dotted line) expansion of the interhemispheric fissure

A consistent pattern that, to the best of our knowledge, has not been previously formally described was seen in 19/83 (23%) of these patients; this will hereafter be referred to as the (Grade 4) massive paramedian injury (MPI) pattern, and we attribute it to an exaggerated form of central hypoxic–ischaemic brain injury. At this end of the spectrum, shown in Fig. 5, we found a diamond-shaped expansion of the interhemispheric fissure, possibly a consequence of nearly complete destruction of the paracentral lobule, SMA and sensorimotor cortex. The parasagittal scores in this subtype were the highest with the average score measured at 13/15. Involvement of the basal ganglia, thalami and hippocampi was noted as a constant association in all 19 MPI patients, with spongiotic cavitation seen involving much of the thalamus and putamen bilaterally, and the hippocampi were always severely injured. Overall white matter volume was reduced, involving the central motor core of the brain and much of the pyramidal cortico-spinal tract as well as greater thinning of the corpus callosum. Secondary ventricular prominence, thinning of the fornix and smaller mammillary bodies were noted as associated features of the MPI pattern. The loss of volume at the brainstem was also found to be more marked in this most severe category of injury, likely a result of Wallerian degeneration.

**Fig. 6:**
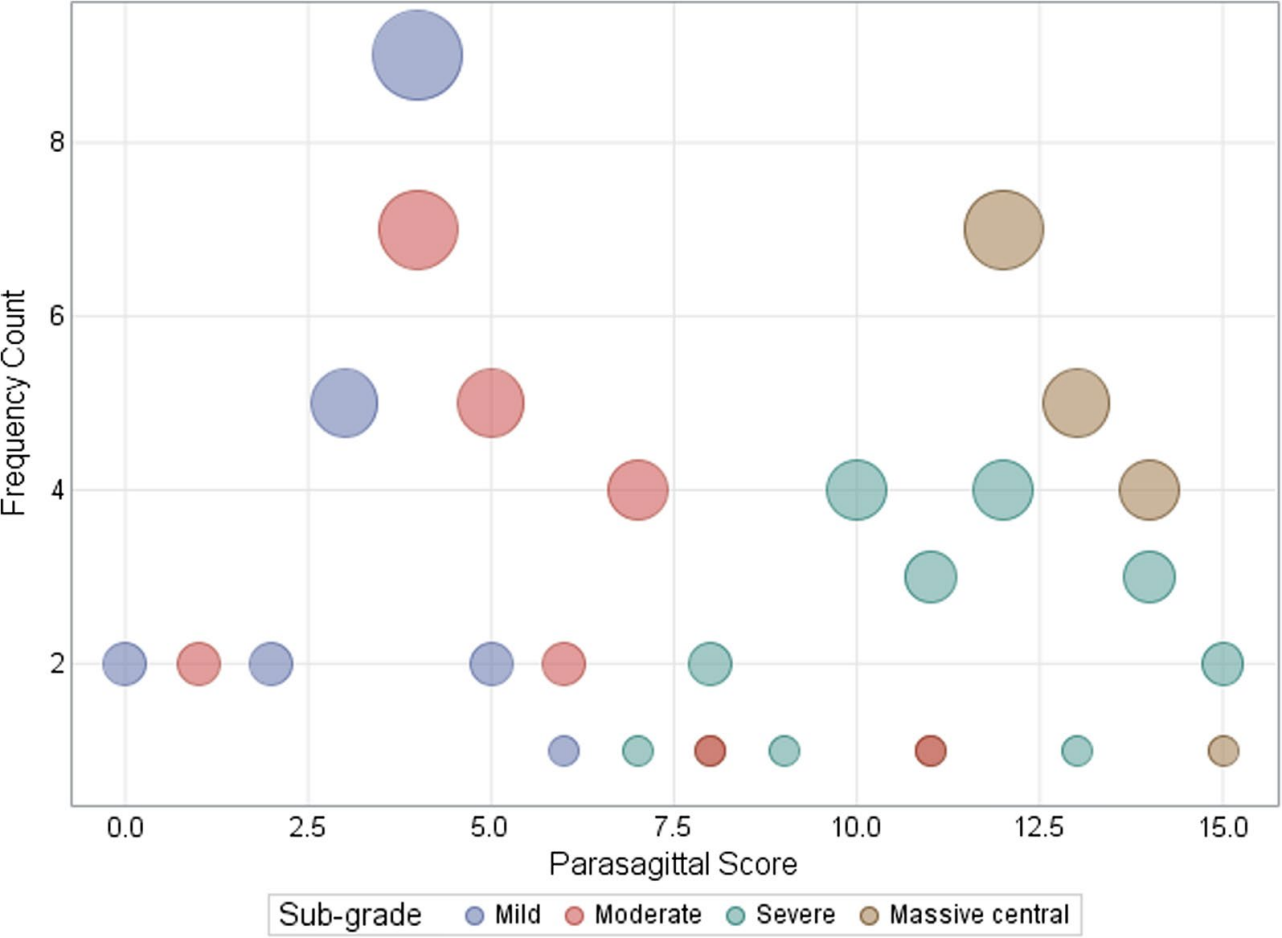
Grid-bubble chart of the frequency of parasagittal scores for subtypes of central injury

The summarised constellation of imaging findings in the four subtypes of central HIBI is categorised in Table 2. Cross-tabulations were made of the injury patterns in each subtype listed in Table 3 and the grid bubble map of Fig. 6 with MPI injury compared to the lower grades of perirolandic injuries (mild, moderate and severe). Note the remarkably higher parasagittal injury scores for the MPI subgroup consequent upon greater degrees of substrate injuries in each of the constituent measured structures.

**Table 3:** Distribution of the parasagittal score between sub-groups

Parasagittal score, *N* (%)	Mild (*N* = 21)	Moderate (*N* = 22)	Severe (*N* = 21)	MPI (*N* = 19)	Overall (*N* = 83)
0	2 (9.5)				2 (2.4)
1		2 (9.1)			2 (2.4)
2	2 (9.5)				2 (2.4)
3	5 (23.8)				5 (6.0)
4	9 (42.9)	7 (31.8)			16 (19.3)
5	2 (9.5)	5 (22.7)			7 (8.4)
6	1 (4.8)	2 (9.1)			3 (3.6)
7		4 (18.2)	1 (4.8)		5 (6.0)
8		1 (4.5)	2 (9.5)	1 (5.3)	4 (4.8)
9			1 (4.8)		1 (1.2)
10			4 (19.0)		4 (4.8)
11		1 (4.5)	3 (14.3)	1 (5.3)	5 (6.0)
12			4 (19.0)	7 (36.8)	11 (13.3)
13			1 (4.8)	5 (26.3)	6 (7.2)
14			3 (14.3)	4 (21.1)	7 (8.4)
15			2 (9.5)	1 (5.3)	3 (3.6)

*P* value < 0.0001

The comprehensive clinical details from the base hospitals were available for 11 of the 19 MPI patients. These 11 patients were selected for detailed evaluation of their MRI findings. The youngest child imaged was 23 months and the oldest was 13 years of age. All 11 patients (six female and five male) were born to Rhesus positive mothers with negative syphilis serology, and all were term neonates; patients 95 and 150 were delivered beyond 40-weeks’ gestation. There were no documented antenatal scan abnormalities or family histories of metabolic disorders. The mother of patient 007 had a history of maternal HIV infection, diagnosed for the first time in this pregnancy, for which antiretroviral therapy was administered. Histories of maternal HIV infections were also known in patients 109 and 229 but they had recorded satisfactory CD4 counts and the appropriate protocols for the prevention of mother to child transmission were followed in each case. Patient 150 had a history of gestational hypertension but no pre-eclampsia and, due to maternal age, an amniocentesis was performed, revealing no chromosomal abnormality. There were no further significant antenatal maternal diseases. Use of epidural anaesthesia in four patients (007, 150, 229 and 297) was associated with maternal hypotension. Severe maternal blood loss during the caesarean section was documented in patient 007, requiring blood transfusion. For this subgroup, we accumulated the available clinical data, which is listed in Table 4 and outlined in Additional file 1 for each child.

**Table 4:** Clinical factors in subset of 11 patients with MPI

Pat Number	G A (weeks)	Age at scan	Gender	A/natal sonar	A/natal risk fact	Prolonged labour	Epidural	Delivery	HIE (grade)	BW (g)	A/S 1 min	A/S 5 min	Vent	Seizures	Postnatal Ultrasound	Post-natal or Follow-up MRI/CT	Other
007	38/40	6	1	Nil performed	HIV, Prior C/S X2 for CPD	1^st^ and 2^nd^ stage	Yes	C/S	3	3000	0	1	Bag and SIMV	Early and severe	Not recorded	Not recorded	Foetal decelerations/severe bradycardia. Prolonged labour followed by delay in C/S (40 min) Severe maternal blood loss during C/S
95	41/40	12	1	Normal 18-week scan	Post-mature Nil else	1^st^ and 2^nd^ stage	No	NVD	3	3500	4	5	Oxygen by NP	Early and severe	U/s on day 15 and at 3 months showed hypoxic–ischaemic brain injury	Not recorded	CPD in a primigravida. Neonatal anaemia required transfusion. LP on Day 5 was normal
109	38/40	6	1	Normal scan performed at local clinic	HIV on antiretroviral therapy	1^st^ and 2^nd^ stage	No	NVD with vacuum	3	4400	6	9	Oxygen by NP	Early and severe	Post-natal ultrasound was normal on day 2	CT scan demonstrated fronto-parietal cortical atrophy at 5 months age	Prolonged second stage with failed vaginal delivery of large baby. Required vacuum extraction with multiple attempts
149	38/40	9	0	Normal scan performed at local clinic	Nil	1^st^ and 2^nd^ stage	No	NVD	3	2500	4	8	Oxygen by NP	Early and severe	Not recorded	CT Scan confirmed hypoxic brain injury with basal ganglia and cortical involvement	CPD in a primigravida Prolonged labour Normal LP
150	41/40	7	1	Normal scan at 25 weeks—by radiologist	Hyper Tension Post-mature	2^nd^ stage	Yes	C/S	3	3800	3	5	SIMV	Early and severe	Normal ultrasound on day 2	Not recorded	Decreased foetal movement, tachycardia and complex variable decelerations. Late Foetal bradycardia, maternal hypotension
177	40/40	7	0	Normal scan performed at local clinic	Prior intra-uterine death	2^nd^ stage	No	NVD With forceps	3	3400	3	5	Oxygen by NP	Early and severe	Not recorded	Not recorded	Severe delay in second stage—140 min. Intravenous Pitocin. Repeated forceps application needed to deliver. Cord found wrapped around neck
190	38/40	9	0	Normal scan performed at local clinic	Nil	1^st^ and 2^nd^ stage	No	NVD	3	3500	3	4	Oxygen by NP	Early and severe	Not recorded	Not recorded	Severe caput and moulding related to CPD
219	41/40	5	0	Normal scan performed at local clinic at 17 weeks	Maternal obesity BMI 45	1^st^ and 2^nd^ stage	No	NVD	3	2860	4	5	NCPAP	Early and severe	Post-natal ultrasound was normal	MRI showed diffuse severe cortical atrophy, white matter loss and corpus callosum thinning	Pitocin augmented delivery. Whole body cooling commenced at 2 h of life, continued for 72 h after delivery. Required intravenous inotropic support
229	40/40	5	0	Normal scan performed at local clinic	HIV positive on antiretroviral therapy	1^st^ and 2^nd^ stage	Yes	C/S	3	2260	2	3	Oxygen by NP	Early and severe	Not recorded	Not recorded	IUGR is suspected given the foetal weight and gestational age. HIV may be associated with chorioamnionitis. Prolonged first and second stage, augmented by oxytocin and epidural used
234	40/40	3	0	Normal scan performed at Clinic at 20 weeks	Nil	2^nd^ stage	No	NVD	3	3430	6	7	Bag	Early and severe	U/S normal after birth. U/S on day 8 showed oedema	CT scan showed no features of metabolic or genetic disorders. Thin Corpus callosum	Prolonged second stage of labour Foetal distress. MSL. Aspiration
297	38/40	2	1	Normal second trimester scan by ob/gynae	Nil	2^nd^ stage	Yes	C/S	3	3130	1	3	Bag	Early and severe	U/S on day 1 was normal. U/S on day 3 showed oedema	MRI on day 15 showed APA changes CT scan at 6 months showed no progressive changes	Prolonged 2^nd^ stage. Foetal distress. Failed forceps extraction. Cephalhematoma. Head cooling done

Regarding gender: 0 = female, 1 = male

*Pat* patient, *GA* gestational age, *A/natal* antenatal, *C/S* caesarean section, *NVD* normal vaginal delivery, *CPD* cephalo-pelvic disproportion, *Vent* ventilation, *MSL* meconium-stained liquor, *SIMV* synchronised Intermittent Mandatory Ventilation, *LP* lumbar puncture, *NCPAP* Nasal continuous positive airway pressure

The common thread with key similarities is listed in Table 5. Common to all these 11 term neonates, was the prolongation of labour, particularly the second stage, often with impaction of the fetus in the birth canal for prolonged periods leading to foetal compromise. Augmentation of labour by oxytocin administration intravenously was noted in the available maternal records. Of note, all the neonates were appropriate (or of slightly increased) weight for gestational age, with normal head circumferences and lengths measured. No neonatal features of metabolic disorders or chromosomal abnormalities were identified. Each child was born after prolonged labour with very low Apgar scores, and grade 3 hypoxic–ischaemic encephalopathy recorded by the attending paediatricians. Severe metabolic acidosis was recorded in each case, with blood gas analyses showing marked negative base excesses. Early and severe neonatal convulsions were noted in each child, requiring phenobarbitone or equivalent anticonvulsant therapy in the immediate or early neonatal period.

**Table 5:** The common findings in all 19 patients with MPI pattern

A common thread…	
Term neonates	
Appropriate or large for gestational age neonates	
Normal antenatal history, all Rhesus positive and syphilis serology negative	
No significant chronic maternal conditions	
Prolonged labour—more often second stage	
Foetal distress, especially with cephalopelvic disproportion	
Epidural anaesthesia with maternal hypotension	
Grade 3 hypoxic–ischaemic encephalopathy	
Low Apgar scores	
Metabolic acidosis	
Neonatal seizures	
Normal immediate postnatal imaging	
Metabolic or chromosomal abnormalities excluded	
Static/non-progressive phenomena on follow-up imaging	
Fairly similar pattern of cerebral injury on imaging	

**Fig. 7:**
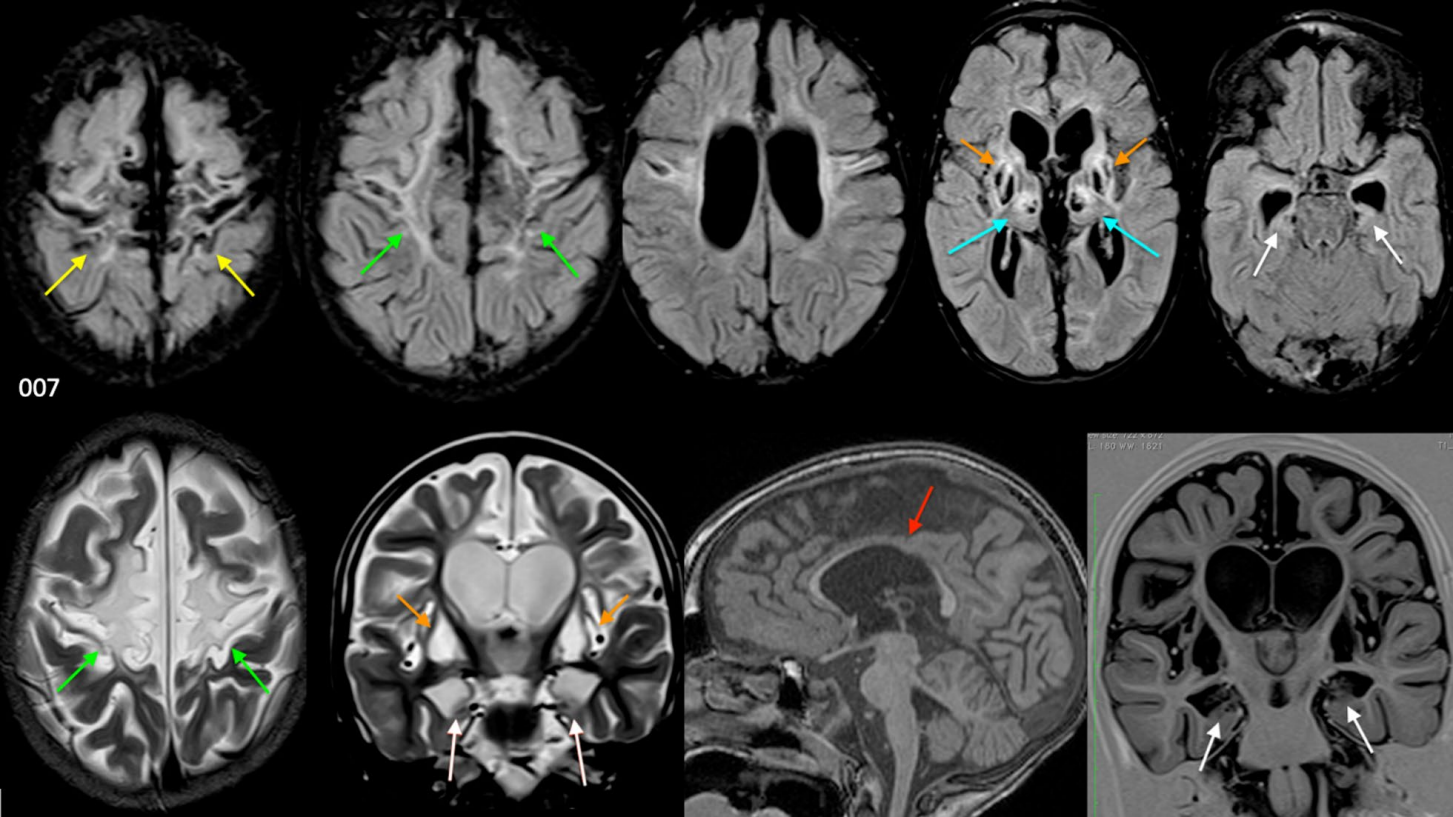
Collage of MR images demonstrating the key features of the massive paramedian injury pattern. Top row = axial FLAIR and bottom row (from left to right) axial T2-weighted, coronal T2-weighted, Sagittal T1-weighted and coronal inversion recovery sequence images. These demonstrate the perirolandic injury (yellow arrows), diamond-shaped expansion of the parasagittal cortex including the paracentral lobule (green arrows), hippocampal destruction (white arrows), putaminal necrosis (orange arrows) and thalamic (blue arrows) cavitation. The red arrow highlights the severe deafferentation thinning of the body and isthmus of corpus callosum

Only two of the 11 infants, both managed at private health care facilities, were treated with cooling (Patient 219 had whole body cooling and patient 297 received head cooling). Postnatal imaging showed no features of major encephaloclastic abnormalities, chromosomal or metabolic disorders in any of the 11 children. There were ultrasound, CT and MRI studies performed in the early and late neonatal period which showed features of HIBI. These changes, though severe, have remained stable in the patients where repeat studies (performed at variable intervals up to several months to years later) are available for comparison. The index patient (Patient 007) selected to showcase the spectrum of findings in the MPI pattern is shown in Fig. 7, with the clinical details summarised in Table 6.

**Table 6:** The summarised clinical details of index patient 007

A 34-year-old Para 3 Gravida 4 mum presented to a level-2 local hospital in early labour. She had two visits to the clinic during her pregnancy and antenatal assessments did not reveal any abnormalities. Her haemoglobin level was normal in the antenatal records. She was diagnosed HIV positive in this pregnancy and had been started on antiretroviral therapy in the third trimester as per the protocol for prevention of mother to child transmission. Her CD4 count was recorded at 521 cells/mm^3^. Prolonged first and second stage of labour was noted with early decelerations annotated on the cardiotocograph. Attempts to allow the labour to progress resulted in further decelerations being noted and a late decision was made to proceed with caesarean section under spinal anaesthesia. The obstetrician reported that the operation was difficult (due to adhesions and scarring of the uterus from two prior caesarean sections) requiring extension of incisions and more than 40 min before extraction of the hypotonic male infant. Maternal hypotension was documented during the delivery. The post-operative maternal haemoglobin level was measured at 7.8 g/dL indicating that the mum suffered severe blood loss. The Apgar scores were calculated as 0/10 at 1 min and 1/10 at 5 min with severe metabolic acidosis (Base excess of − 11.8) on cord blood gas. The child required intubation and ventilation. The neonatal blood glucose levels and other electrolytes were normal. Unfortunately, due to the local lack of expertise and imaging services, there was no early neonatal imaging available. MRI was performed at the age of 6 years. See Fig. 2	

**Fig. 8:**
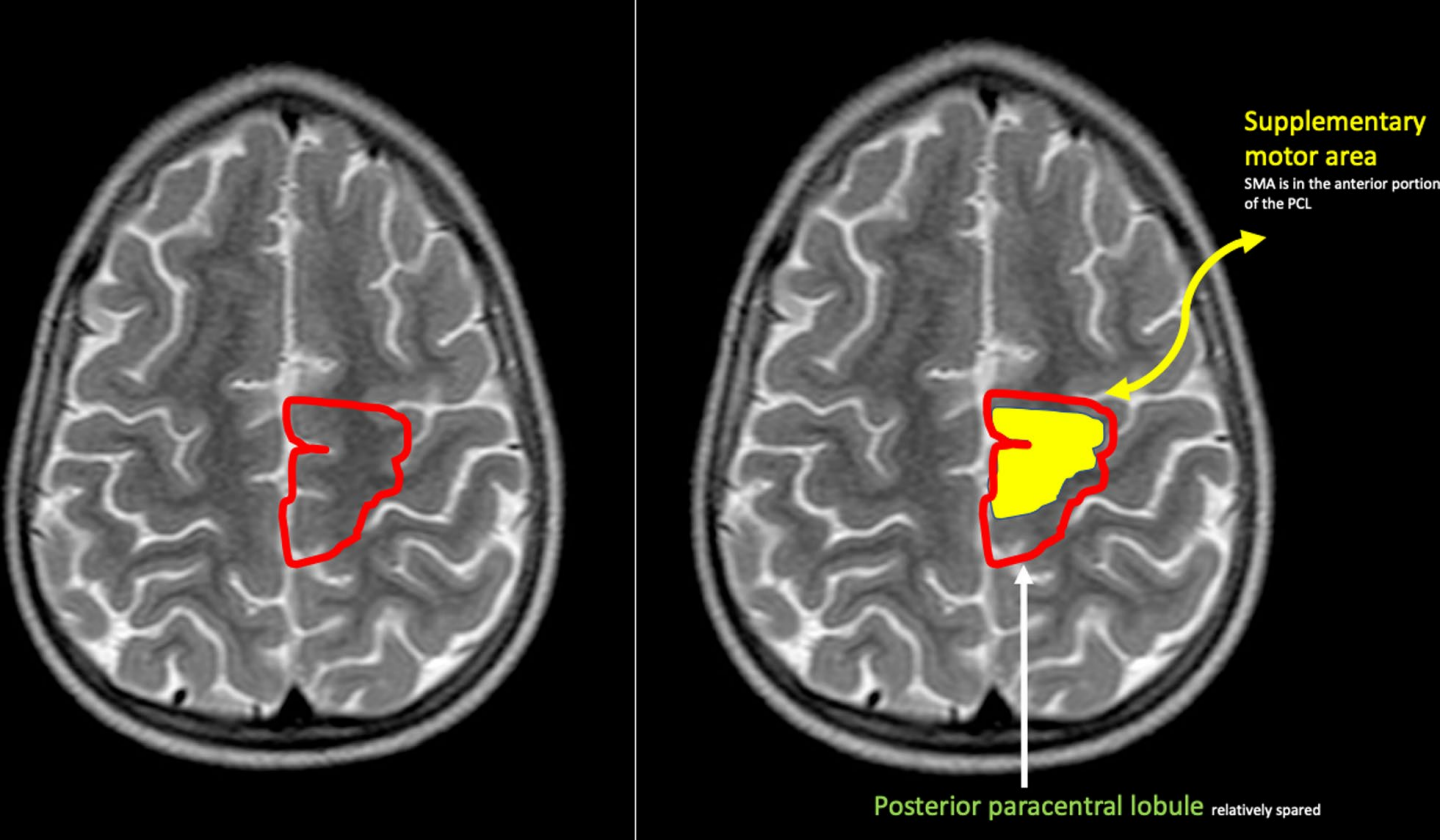
The location of the paracentral lobule (PCL) with the anterior PCL connecting the supplementary motor area (SMA) to the precentral primary motor cortex and the posterior PCL connecting to the postcentral gyrus

In this most destructive Grade 4 subtype of parasagittal perirolandic HIBI, which we have termed as the *massive paramedian injury* subtype, there is complete paracentral lobule destruction (Figs. 5, 7, 8), which is a key and constant feature identified in all 19 cases. The widening of the IHF now extends further anteriorly, with superior frontal gyrus also involved opening an inverted V-shape anteriorly. There is also variable posterior extension of IHF widening beyond the paracentral lobule towards the ventral aspect of the SPL, which results in a smaller V-shape posteriorly. Figure 5 shows the anterior inverted V-shape and posterior V-shape that combine to form a diamond-shaped expansion of the perirolandic interhemispheric region. Significant collapse of the central cerebral core, consisting predominantly of motor connections, is noted to extend through the centrum ovale, cortico-spinal tracts, thalami and putamina associated with remarkable secondary atrophy of the corpus callosum and hippocampus. In addition, these children suffer extensive white matter volume loss, with relative sparing of the cortex (except for the calcarine region, the insular cortex and Heschl gyrus) in most of the rest of the cerebrum. Smaller substrate injuries of the fornix and mammillary body are also noted consistently in conjunction with hippocampal destruction in this subtype. Of note, is the observation that all these MPI cases were associated with a severe and sustained perinatal complication precipitating the hypoxic–ischaemic insult.

The comparison of the MPI subtype to the rest of the lesser subtypes of central injury (grouped together) is depicted in Table 7. Cross-tabulations of the patients with less severe substrate injuries (including IHF widening, paracentral lobule scores and the precentral and post-central gyrus injuries) show statistically significant difference compared to those in the MPI subgroup, with *p* values < 0.001. Statistically significant differences were also found in the injuries identified in the hippocampi (*p* = 0.006), thalami (*p* = 0.002), putamina (*p* < 0.01), and insular cortex (*p* = 0.008). The odds of experiencing injuries to the parasagittal structures, enumerated in Table 8, confirms the higher propensity for these injuries in the MPI subgroup; the odds of massive paramedian injury in the less widened IHF was found to be 0.08 times less likely than for severely widened interhemispheric fissure (OR: 0.08, 95% CI 0.02–0.25, *p* < 0.001). Similarly, massive paramedian injury in the uninvolved pre-central gyrus is 0.02 times less likely than for severely destroyed pre-central gyrus (OR: 0.02, 95% CI 0.00–0.14, *p* < 0.001).

**Table 7:** Key features of each subtype of parasagittal central hypoxic–ischaemic injury

Substrate injury		Mild/Mod/Sev (*N* = 64)	MPI (*N* = 19)	Overall (*N* = 83)	*P* value
IHF Widening, *n* (%)	Other	6 (31.6)	55 (85.9)	61 (73.5)	< 0.001
	Severe	13 (68.4)	9 (14.1)	22 (26.5)	
Pre-central Gyrus Score, * n* (%)	Other	1 (5.3)	49 (76.6)	50 (60.2)	< 0.001
	Severe	18 (94.7)	15 (23.4)	33 (39.8)	
Post-Central Gyrus Score, * n* (%)	Other	4 (21.1)	52 (81.3)	56 (67.5)	< 0.001
	Severe	15 (78.9)	12 (18.8)	27 (32.5)	
SFG Score, * n* (%)	Other	18 (94.7)	62 (96.9)	80 (96.4)	0.547
	Severe	1 (5.3)	2 (3.1)	3 (3.6)	
Paracentral Lobule Score, * n* (%)	Other	1 (5.3)	45 (70.3)	46 (55.4)	< 0.001
	Severe	18 (94.7)	19 (29.7)	37 (44.6)	
Thalamus Score, * n* (%)	Other	3 (15.8)	37 (57.8)	40 (48.2)	0.002
	Severe	16 (84.2)	27 (42.2)	43 (51.8)	
Putamen Score, * n* (%)	Other	1 (5.3)	38 (59.4)	39 (47.0)	< 0.001
	Severe	18 (94.7)	26 (40.6)	44 (53.0)	
Heschl Gyrus, * n* (%)	Other	15 (78.9)	60 (93.8)	75 (90.4)	0.076
	Severe	4 (21.1)	4 (6.3)	8 (9.6)	
Insular Cortex, * n* (%)	Other	13 (68.4)	60 (93.8)	73 (88.0)	0.008
	Severe	6 (31.6)	4 (6.3)	10 (12.0)	
Hippocampus Score, * n* (%)	Other	2 (10.5)	30 (46.9)	32 (38.6)	0.006
	Severe	17 (89.5)	34 (53.1)	51 (61.4)	
Cerebellum, * n* (%)	Normal	12 (63.2)	47 (73.4)	59 (71.1)	0.400
	Involved	7 (36.8)	17 (26.6)	24 (28.9)	

*IHF* interhemispheric fissure, *SFG* superior frontal gyrus, *other* lower grades of substrate injuries, *Mod* moderate subtype, *Sev* severe subtype, *MPI* massive paramedian injury

**Table 8:** Factors associated with MPI compared to other subtypes with reference being the severe grade of substrate injuries

Feature	OR	95% CI	*P* value
IHF Widening	0.08	0.02–0.25	< 0.001
Pre-central gyrus	0.02	0.00–0.14	< 0.001
Post-central gyrus	0.06	0.02–0.22	< 0.001
SFG Score	0.58	0.05–6.77	0.664
Paracentral lobule	0.02	0.00–0.19	< 0.001
Thalamus	0.14	0.04–0.52	0.003
Putamen	0.04	0.00–0.30	0.002
Heschl Gyrus	0.25	0.06–1.12	0.070
Insular cortex	0.14	0.04–0.59	0.007
Hippocampus	0.13	0.03–0.63	0.011

*OR* odds ratio, *95%CI* 95% confidence interval, *IHF* interhemispheric fissure, *SFG* superior frontal gyrus

## Discussion

Kinney and Volpe categorised the patterns of HIBI into 3 major subgroups; diffuse, cerebral cortex-deep nuclear and deep nuclear-brainstem [12]. These categories of HIBI have been renamed and subclassified by others over the last three decades [2–6, 8]. The classification used in this study is outlined in Table 1 [8]. Parasagittal cortex injuries sustained during perinatal hypoxia–ischaemia have been described by several authors and have largely been attributed to partial prolonged or watershed territory ischaemia [7]. In such cases, the areas of involvement have been divided into the anterior watershed, perisylvian watershed and posterior watershed territories [13]. We have shown in our collection of cases that parasagittal central type HIBI can sometimes be identified in more severe sustained hypoxia–ischaemia. This is most notable when a severe reduction in perfusion of the high metabolic zones, in the motor, supplementary and motor association regions of the frontal cortex is sustained over a prolonged period, with inadequate capacity for the autoregulatory mechanisms to redirect perfusion to these areas [8, 14].

**Fig. 9:**
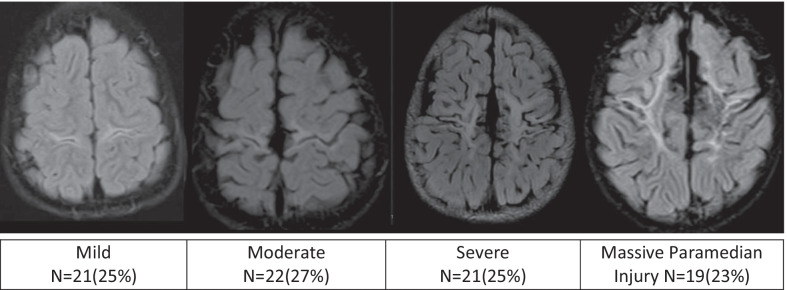
The proposed classification of parasagittal perirolandic central type injury

Based on the findings of this study our proposed classification of the subtypes of central injuries is shown in Fig. 9. In all four subtypes, there is differential involvement (but more consistent in the MPI group compared to severe subgroup as per odd’s ratios in Table 8) of the deep nuclei, the primary visual cortex, the cerebral white matter, corpus callosum, hippocampi and smaller substrates including the mammillary bodies, fornix, insular and heschl gyrus. This results from the fact that less severe reduction in energy substrate causes injury mostly affecting areas with higher metabolic demands (usually the most mature areas); as the reduction in energy substrate becomes more severe, more areas are involved. Progressive destruction was also noted of the insular cortex and Heschl gyrus from the mild to more severe subtypes, congruent to the perirolandic, putaminal and thalamic injuries. Variable deafferentation thinning of the corpus callosum, especially of the body and isthmus, was noted correlative to the perirolandic sensorimotor cortex destruction.

In some instances, the injury may be very subtle or limited to the sensorimotor strip with little or no widening of the central sulcus; we propose that this should be called the *mild perirolandic (Grade 1) injury* subtype. The second subtype is the *moderate perirolandic (Grade 2) injury* group with paracentral lobule and sensorimotor cortex injury, resulting in some widening of the interhemispheric fissure. In the third subtype, called the *severe perirolandic (Grade 3) injury* subtype, there is extension of the injury to the margins of the paracentral lobule and the lateral edges of the central sulcus; the associated atrophy in these regions is more marked, with greater FLAIR hyperintensity in the subcortical white matter.

In the *MPI (Grade 4)* subtype, there is broad widening of the interhemispheric fissure secondary to near complete destruction of the paracentral lobule, with resultant diamond-shaped excavation of the central core of the cerebrum, including part of the centrum ovale. This injury extends towards the superior frontal gyrus (anteriorly) and variably towards the superior parietal lobule (posteriorly). This Grade 4 subtype is the most destructive parasagittal central motor core injury, which we propose naming as the *massive paramedian injury*. The pattern approaches but does not equate to the described pattern of “near total brain injury” [4] or “acute near total asphyxia” [14], many of whom may not proceed to be imaged, succumbing to early neonatal demise.

Asphyxia is the pathophysiological result of metabolic acidosis following hypoxia and hypercapnia [15]. Several studies have attempted to recreate the clinical scenario mimicking a perinatal sentinel event or more prolonged complicated labour leading to HIBI, in order to analyse the outcome by measuring the degree of brain injury and characterising specific patterns of injury that may be attributed to each scenario. Models depicting total brain injury typically include hypoxia–ischaemia that will result in an oxygen saturation level of 30%, a bradycardia with 50% reduction in heart rate, and a near complete cessation of blood pressure [15]. The critical threshold of the duration of injury (after which (1) substrate changes are more likely to be apparent on imaging and (2) long-term adverse outcomes ensue), is estimated at 10 min [4, 16]. All the patients that we classified with the MPI subtype of central injury had suffered marked prolongation of labour, particularly the second stage.

Early studies [17–20] in primate and sheep models provide invaluable insight into the pathogenesis of perinatal hypoxic–ischaemic brain injury. The duration and severity of the hypoxic episode are critical to the eventual outcome. Perhaps the closest simulated correlate to the pathology presented here in instances of MPI, is the 30-min experiment by Gunn et al. [20]. When the carotid arteries of near-term sheep fetuses were occluded for a single episode of 30 min duration, severe cortical neuronal loss was found. In contrast, less severe corpus striatum injury was seen when the sentinel events, lasting 10 min each, were repeated 3 times at intervals of 1 or 5 h. The combination of perirolandic, basal ganglia, thalamic and other high metabolic substrate injuries shown in our MPI group closely approximates the injuries of the Gunn experiment. This corresponds in vivo with prolongation of the second stage of labour (a feature identified in all patients with MPI) and consequent severe, sustained hypoxic-ischaemic insult to the susceptible fetus.

In a landmark article of our time, Barkovich [1] described the pattern of cerebral injury identified on CT and MR imaging in neonates that suffered profound asphyxia. The injuries sustained were thought to be mediated by some common biochemical/neurotransmitter (glutamate/NMDA) receptor propensity at the affected brain areas during a period of active myelination of these areas, causing increased susceptibility to hypoxic–ischaemic brain injury [21]. To better understand the patterns of injury sustained in profound asphyxia, it is important to clarify the location, function and structure of these receptors, which are believed to play a key role in mediating the cerebral injury. Glutamate-mediated injury is high in the basal nuclei, particularly the putamen, the thalami and perirolandic neurons, largely due to the overactivation of excitatory amino acid receptors [22]. NMDA receptors are proteins located in the post-synaptic neurons where they are embedded into the cell membranes. These are an important component of the signal transduction pathways that control opening and closing of ion channels; they are one of three so-called ionotropic receptors {viz NMDA, α-amino-3-hydroxy-5-methylisoxazole-4-propionic acid (AMPA), and kainic acid (KA)}. NMDA receptors are responsible for mediation of injury to the neurons in several structures (including the cerebral cortex, hippocampus, basal nuclei and thalamus) associated with a cascade of cellular events leading to hypoxic–ischaemic injury in multiple animal models [23]. There is also growing research into the use of anti-NMDA receptor agents in the neuroprotection of neonates at risk of hypoxic–ischaemic brain injury [24]. For these reasons we studied, in detail, the collective injuries of these specific substrates in central type HIBI.

Neuropathological correlation of the magnetic resonance imaging features with pathological findings confirms that the histopathological substrate of cases of profound hypoxic–ischaemic encephalopathy from mild forms to massive paramedian injury entails an encephaloclastic (destructive) lesion of the brain [9]. The brain imaging counterparts for these pathological processes are: T2/FLAIR hyperintensities representing neuropil spongiosis, brain matter atrophy representing cell death and the “luxury perfusion” phenomenon representing secondary micro-vascular proliferation. Historically in the human body, there are two types of cell death described: unprogrammed cell death (necrosis) and programmed cell death (apoptosis, necroptosis, etc.). In the central nervous system, a third type of cell (neuronal) death is described: neurotransmitter excitotoxicity involving glutamate and gamma aminobutyric acid (GABA) [9]. The hypoxic–ischaemic insults may not cause immediate neuronal death (primary cell death), but may precipitate a complex series of biochemical events leading to cell death many hours, or even days, later (secondary cell death). The primary cell death is represented by necrosis and programmed cell death, while the secondary cell death is manifested as neurotransmitter excitotoxicity [25].

The central nervous system in general, and the cerebral cortex in particular, are systems that evolve principally for controlling the animal motor activity [9]. The frontal cortex, which includes the primary motor area and the supplementary motor areas, is the most structurally and functionally complex region of all the neocortex; it is also, however, highly susceptible to different kinds of insults, including hypoxic–ischaemic injuries [9]. This complexity of the motor cortex, together with the high metabolic demands of the large local pyramidal neurons, explains the reason for the increased T2/FLAIR signal in the perirolandic strip in cases of profound ischaemia. There is an innate neural link between these high metabolic areas. From our analysis of the substrate injuries, we have shown definitive MRI features that attest to this network linking the high metabolic areas. In particular, the links between the Rolandic sensorimotor cortex, SMA of the PCL, hippocampi and basal ganglia are most important [26].

A common component of the parasagittal cerebral cortex substrate that links the mild to the more severe subtypes of profound parasagittal central injuries is the involvement of the paracentral lobule (PCL). This structure, shown in Fig. 8, is continuous with the motor cortex anteriorly and the sensory cortex posteriorly. Injuries involving the PCL lead to variable degrees of widening of the IHF at the medial margin of the central sulcus. There are several neural networks that connect the PCL, particularly the supplementary motor area, to components of the extra-pyramidal system including the posterior putamina and secondarily the thalami [27, 28]. These neural connections have been well demonstrated on diffusion tensor imaging by Lehericy et al. [29]. It has also been shown that the SMA is unlike other cortical motor areas in having three to four times more basal ganglia input than cerebellar input [9], confirming this complex neural network involved in motor processing [26, 30]. We have shown this to be a key structure, (statistically significant with *p* value < 0.001), in incremental grades of perirolandic injury, progressively destroyed with widening of the interhemispheric fissure, thereby forming an important component of our grading system from mild to severe central injury leading up to the MPI subtype. Table 7 confirms the significance of the structural (especially PCL) injuries in the MPI subtype when compared to the lesser subgroups combined. The odds of massive paramedian injury in the less injured PCL is 0.02 times less likely than for severely injured PCL (OR: 0.02, 95% CI 0.00–0.19, *p* < 0.001).

There is secondary neural connectivity between the cortex and striatum to the insular cortex, the Heschl gyrus and hippocampus [31]. Our study highlights this intricate neural network showing the connectivity between the cerebral cortex, the putamen and thalamus with secondary substrates including the STN, Heschl gyrus, insulae and hippocampi all demonstrating selective neuronal necrosis when the high metabolic areas suffer severe, sustained hypoxia–ischaemia. The hippocampal involvement in severe ischaemia is probably related to the fact that pyramidal neurons of this area have a large number of glutamate receptors, including NMDA, AMPA, kainate and metabotropic receptors. [9] The mechanism of death in these neurons is classically neurotransmitter excitotoxicity. The glutamate storm in the hippocampal pyramidal neurons explains the seizure activity in most of these patients, and we have shown hippocampal destruction is a consistent injury in all (100%) cases of MPI. Lesser degrees of hippocampal injury were still identified in the lower grades of central HIBI. Hence, this substrate is closely linked to the RBGT pattern of injury as described above.

A limitation of this study is the retrospective accumulation of data, lack of availability of clinical and biochemical records for many neonates, especially in resource-constrained settings. This has resulted in the smaller cohorts in the subgroups of central injury described here. Perhaps with larger multicentre collaboration and better prospective data collection, these patterns may be better analysed and described.

## Conclusion

Here, we have presented a new perspective with a gradation of the parasagittal cortex injury in children who have suffered severe and sustained hypoxic–ischaemic insults. We propose that this four-tiered gradation correlates with the severity of the perinatal insult and the attendant secondary and subsequent cascades of cellular injury. In this new classification, we have also introduced the most severe subtype of this injury which we have termed the *massive paramedian injury* in which the entire central motor core of the brain is destroyed. Common clinico-pathological features have been identified with the MPI subtype in all the cases presented here, and in particular, these were all associated with prolongation of the second stage of labour.

## Supplementary Information

**Additional file 1**. Supplementary clinical details and images of the additional 10 MPI patients.

## Data Availability

The data that support the findings of this study are available on the PACS archive of Lake Smit and Partners Inc., but restrictions apply to the availability of these data, which were used under license for the current study, and so are not publicly available. Data are however available from the authors upon reasonable request and with permission of patients and Lake Smit and Partners Inc.

## Abbreviations

AMPA: α-Amino-3-hydroxy-5-methylisoxazole-4-propionic acid
BA4: Brodmann area 4
BA6: Brodmann area 6
BGT: Basal ganglia–thalamus
GABA: Gamma aminobutyric acid
HIBI: Hypoxic–ischaemic brain injury
IHF: Interhemispheric fissure
KA: Kainic acid
MPI: Massive paramedian injury
NMDA: N-methyl-D-aspartate
PCL: Paracentral lobule
RBGT: Rolandic-basal ganglia–thalamus pattern
SAS: Statistical Analysis Software
SFG: Superior frontal gyrus
SMA: Supplementary motor area
SNr: Pars reticularis of substantia nigra
SPL: Superior parietal lobules
STN: Subthalamic nucleus
