# Supplementary material for: A proposed magnetic resonance imaging grading system for the spectrum of central neonatal parasagittal hypoxic–ischaemic brain injury
Source: Insights Imaging. 2022 Jan 24;13:11. doi: 10.1186/s13244-021-01139-7 (PMC8787015; doi:10.1186/s13244-021-01139-7)
Supplement: Supplementary file 1 — Additional file 1. Supplementary clinical details and images of the additional 10 MPI patients. [file 13244_2021_1139_MOESM1_ESM.pdf]

ELECTRONIC SUPPLEMENTARY MATERIAL

Supplementary clinical details and images of the additional 10 MPI patients

Patient 95

A 29-year-old primigravida, was followed up through her pregnancy at a local district clinic until term. The mother was treated for urinary tract infection during the pregnancy. There were no further risk factors identified. She presented after term at 41-weeks gestation with a difficult and prolonged labour. According to the patient, the attending midwife advised her to try to force the baby out but this was not possible due to cephalo-pelvic disproportion. After more than 2 hours, a male infant with a birth weight of 3500g was delivered vaginally. The Apgar scores were noted to be low (4/10 and 5/10) with no cry documented at birth. A severe metabolic acidosis was noted with a base excess of 22. The neonatal blood glucose level was recorded at 4.3mmol/L. The neonate was floppy and required oxygen supplementation. No ventilation or cooling was performed. An ultrasound scan was performed on day 18 and this demonstrated superior periventricular white matter changes attributed to hypoxic ischemic injury. The ventricles were normal in size and there were no surface collections or posterior fossa abnormalities noted. A follow-up ultrasound at 3 months' age showed evolving encephalomalacia in the frontoparietal area with mild prominence of the lateral ventricles as a secondary phenomenon. MRI study was performed at 11 years of age and perirolandic distribution of cerebral abnormalities with slightly more atrophy and basal ganglia changes were noted. See Figure 10.

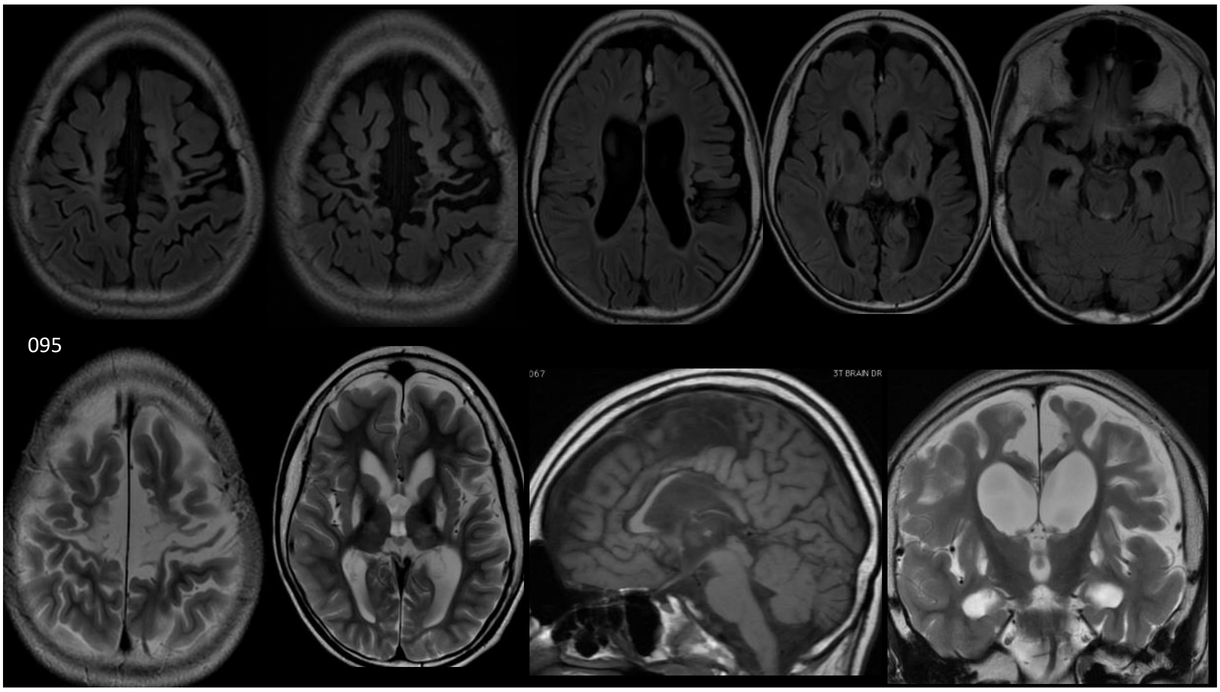

Figure 10 Collage of MRI images obtained for patient 095

Patient 109

The mother, a 35-year-old Para 4 Gravida 5, presented in labour at term (38 weeks-gestation) to the local clinic. She had been following up at the same clinic through her pregnancy. Her HIV status was positive, and she had been on dual antiretroviral therapy through the pregnancy. Her haemoglobin level was normal at 12.3g/dL. There were no further risk factors identified She was transferred to the regional hospital for management of the delivery. The fetal CTG was not well recorded. Prolonged second stage of labour was noted with the fetus impacted within the birth canal for 20 minutes before augmentation of vaginal delivery by vacuum extraction. Two applications of the vacuum were required before the fetus was delivered. Birth weight was 4400g. The Apgar scores were recorded as 6/10 and 9/10 with no cry documented at birth. The liquor was mixed with thick meconium and the neonate required repeated suctioning of the airways. The neonate was floppy and his blood glucose level was found to be 8.4mmol/L. He did not require ventilation; oxygen supplementation was provided by nasal prongs. No cooling was performed. An early ultrasound showed normal appearing brain with no congenital abnormalities or intracranial haemorrhage. A CT scan performed at 5 months of age showed features of hypoxic ischemic brain injury and no evidence of metabolic disorder or leukodystrophy. MRI study was performed at 6 years of age with perirolandic, and basal ganglia changes similar in distribution to those seen on the prior CT Scan. See Figure 11.

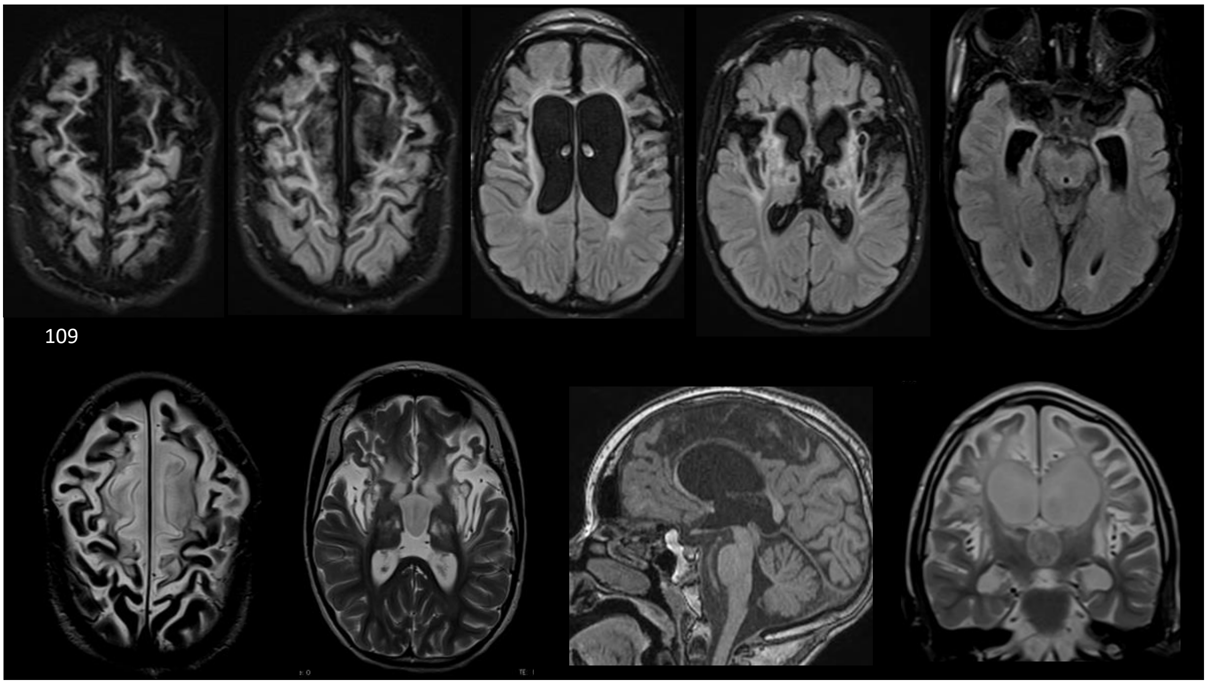

Figure 11 Collage of MRI images obtained for patient 109

Patient 149

The mother, a 22-year-old primigravida, presented to a local district Level 1 clinic at term. She had been following up at the same clinic through her pregnancy with no risk factors identified. An antenatal scan performed in the second trimester was normal. Prolongation of the latent phase of labour was documented by the attending midwife, related to cephalo-pelvic disproportion. Limited intrapartum resuscitation was available at the clinic and the fetal condition was not recorded during the second stage until vaginal delivery of the female infant. Birth weight was 2500g, head circumference was 34cm and length 49cm. The Apgar scores were noted to be low (4/10 and 8/10) with no cry documented at birth. Neonatal convulsions were recorded, and anticonvulsant therapy was initiated. The hypotonic neonate was not ventilated but required oxygen supplementation. No cooling was performed. A few hours after delivery, the neonate was transferred to a Level 2 facility where neonatal care was available. Seizures however continued and after a day, she was referred to a tertiary level hospital for further management. A CT scan performed confirmed hypoxic ischemic brain injury and no evidence of metabolic disorder or leukodystrophy. Follow up CT scan done at 3 months did not show any progressive brain destruction. MRI study was performed at 9 years of age and there was a similar distribution of cerebral abnormalities with only slightly more atrophy, but no new lesions demonstrated. See Figure 12.

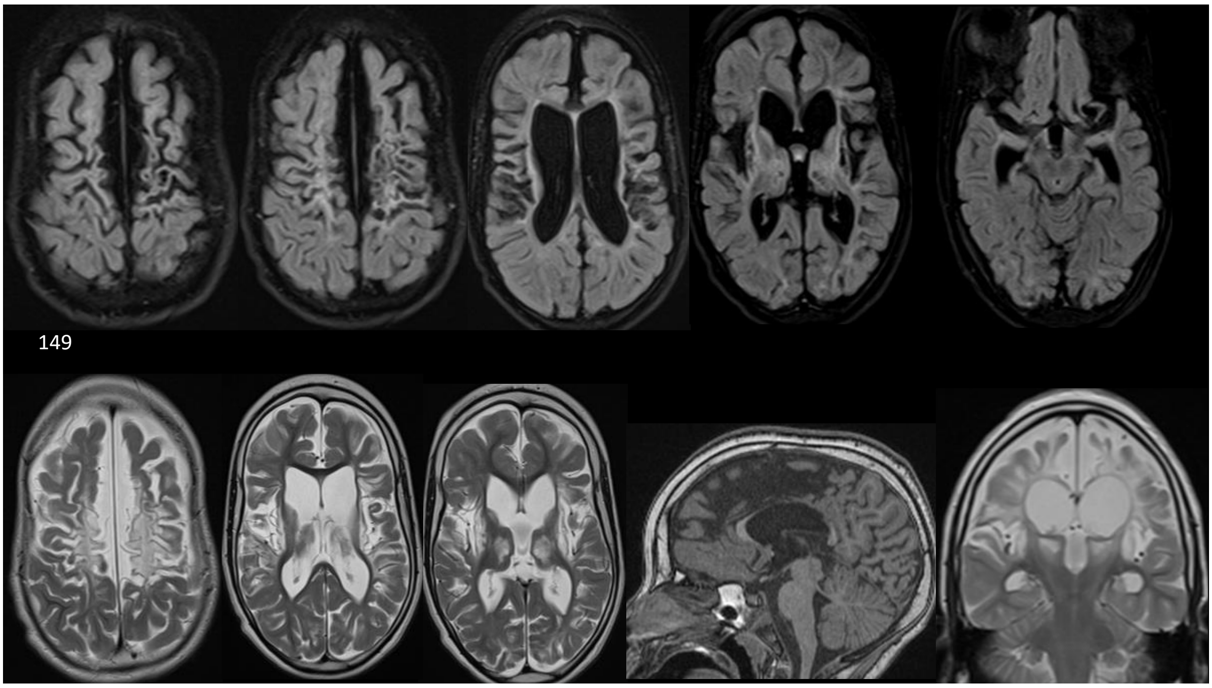

Figure 12 Collage of MRI images obtained for patient 149

Patient 150

A 36-year-old Para 1 Gravida 2 mother with background history of systemic hypertension, presented in labour at term. She had been followed up by her private obstetrician throughout her pregnancy with no additional risk factors identified. An antenatal scan performed in the second trimester at 18-weeks' gestation was normal. Amniocentesis was performed at this gestation due to maternal age. This did not reveal any chromosomal abnormality. Her haemoglobin level was normal (12.6g/dL). Follow-up ultrasound performed by a radiologist at 25-weeks' gestation showed normal fetal development with no structural abnormalities. There was prolongation of the latent and active phases of the first stage of labour. An epidural anaesthetic was administered prior to proceeding with caesarean section. Severe maternal hypotension and fetal bradycardia were documented after epidural infusion started. A hypotonic male child was extracted approximately 86 minutes after fetal bradycardia was demonstrated on cardiotograph. The child did not cry at birth and reduced Apgar scores were noted of 3/10 and 5/10 at 1 and 5 minutes respectively. Birth weight was 3800g. The neonate was intubated and ventilated. Anticonvulsant therapy was initiated after repeated seizures. No cooling was performed. The child was transferred to another hospital for further intensive neonatal care. An ultrasound on day 2 was found to show normal brain volume with no features to indicate congenital or chromosomal abnormality. MRI study was performed at 7 years of age. See Figure 13.

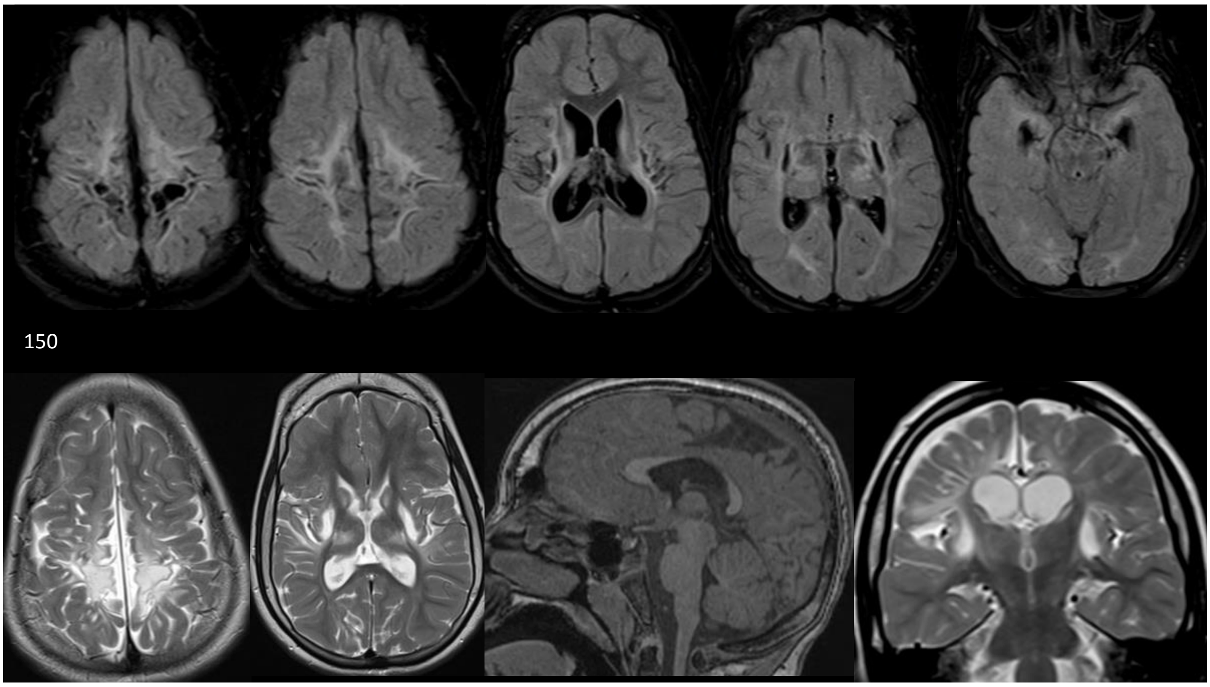

Figure 13 Collage of MRI images obtained for patient 150

Patient 177

A 23-year-old mother was followed up through her pregnancy at a local district clinic until term. She had a background history of a prior pregnancy 6 years earlier with a poor outcome of intrauterine death at near term. This was followed by a second normal pregnancy with a favourable outcome. In this pregnancy, the mother had been well. There were no further risk factors identified. An antenatal scan performed in the second trimester was normal at 18 weeks gestation. She presented at term in the latent phase of labour. The membranes were artificially ruptured and the rest of the first stage was documented as being normal however cardiotocograph and other recordings were scanty in the available notes. There was severe prolongation of the second stage of labour. The initial attempt at normal vaginal delivery was unsuccessful. The foetus was noted to be impacted in the vaginal canal and two subsequent attempts were made to deliver using forceps. 140 minutes after initiation of the second stage of labour, a female infant with a birth weight of 3400g, head circumference of 36cm and length 48cm was delivered vaginally. The umbilical cord was found to be tightly wrapped around the neck. The Apgar scores were noted to be low (3/10 and 5/10) with no cry documented at birth. Neonatal convulsions were recorded, and anticonvulsant therapy was initiated. The neonate was floppy and required oxygen supplementation. No ventilation or cooling was performed. Postnatal imaging was not recorded. MRI study was performed at 7 years. See Figure 14.

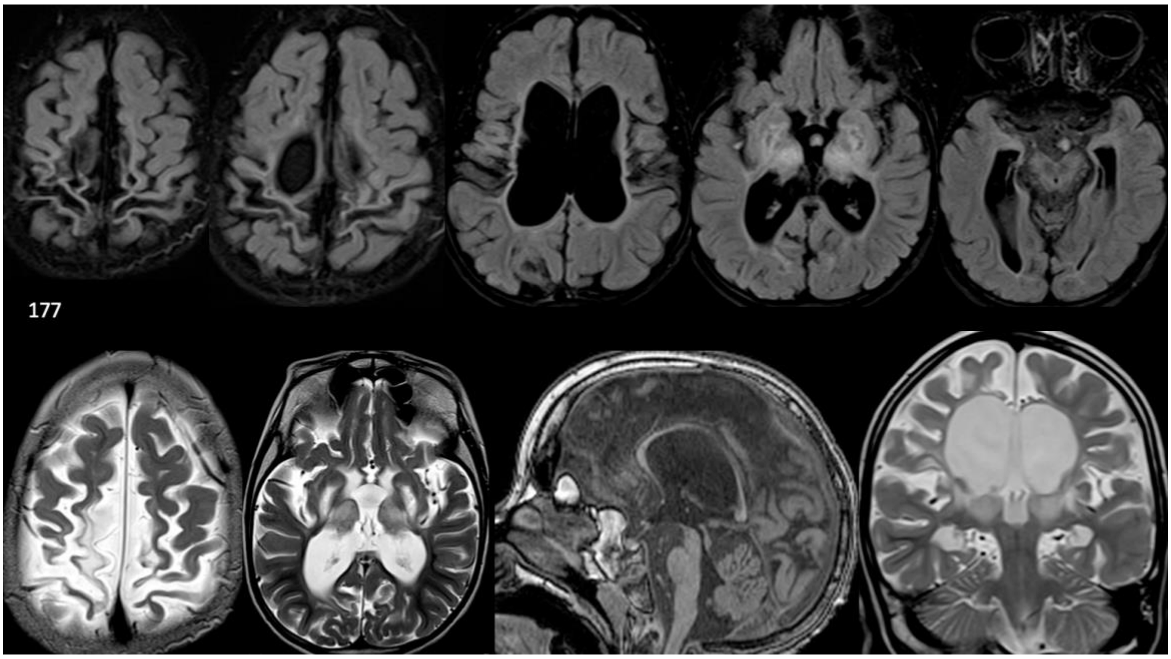

Figure 14 Collage of MRI images obtained for patient 177

Patient 190

A 23-year-old Para 1 Gravida 2 mother with no background medical or family history had 5 antenatal visits at her local hospital. An antenatal sonar performed in the second trimester at the obstetric department was normal. Her haemoglobin level was 10.1g/dL, for which she was prescribed haematinics. There was prolongation of the first and second stages of labour. A hypotonic female child was delivered by vaginal delivery, augmented by an episiotomy. The child did not cry at birth and reduced Apgar scores were noted of 3/10 and 4/10 at 1 and 5 minutes respectively. Birth weight was 3500g, the head circumference was 35cm and the neonatal length 50cm. Blood glucose level was 9.6mmol/L. Severe caput succedaneum and moulding of the cranium were noted, confirming prolonged impaction in the birth canal due to cephalo-pelvic disproportion. Early neonatal seizures required anticonvulsant therapy. No cooling was performed. There are poor clinical records available and post-delivery imaging reports are not available. The paediatrician did not find any clinical/ biochemical indicators of metabolic disorders. An MRI study was performed at 7 years of age. See Figure 15.

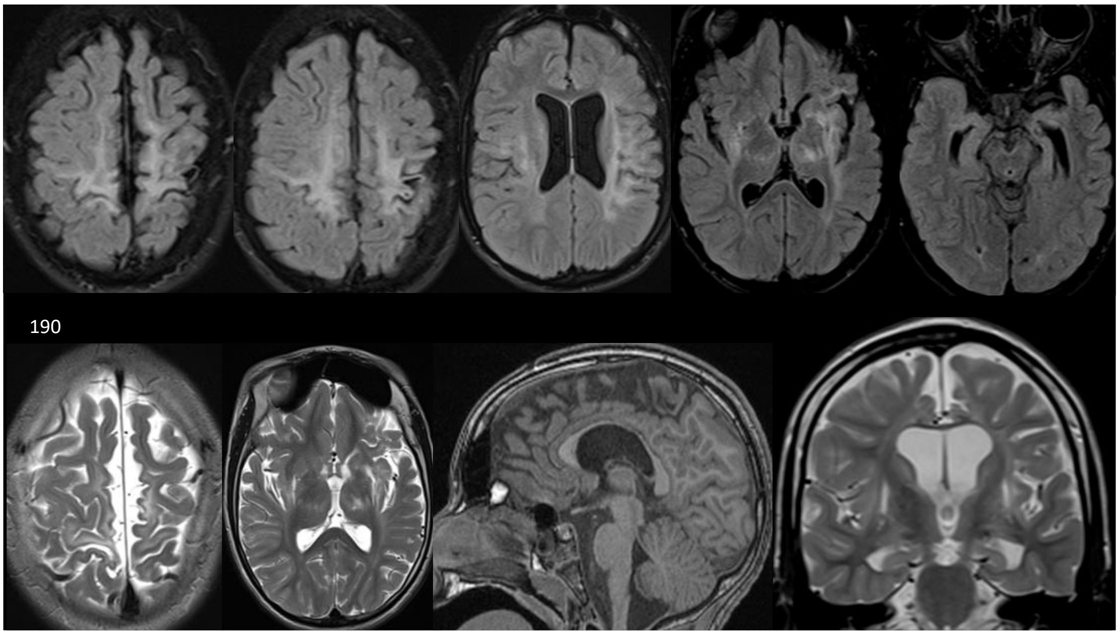

Figure 15 Collage of MRI images obtained for patient 190

Patient 219

A 29-year-old primigravida, with no medical or family history of note, was followed up through her pregnancy at a local district clinic until she presented at 40 weeks and 5 days gestation. The mother was obese with a BMI of 45. An antenatal scan performed in the second trimester was recorded as normal at 18-weeks' gestation. She underwent a difficult and prolonged labour. After several hours of labour, augmented by intravenous oxytocin, she delivered by vaginal delivery, a female infant with a birth weight of 2860g, head circumference of 36cm and length 44cm. The Apgar scores were recorded to be low (4/10 and 5/10) with no cry documented at birth. A severe metabolic acidosis was noted with a base deficit of -14.5. The neonatal blood glucose level was recorded at 6.5mmol/L. The hypotonic child required assisted ventilation by NCPAP and whole-body cooling was instituted. Neonatal convulsions were noted on day 2 and anticonvulsant therapy was initiated. An ultrasound scan was performed while in ICU and this demonstrated periventricular white matter changes attributed to hypoxic ischemic injury. The ventricles were normal in size and there were no surface collections or posterior fossa abnormalities noted. An MRI study was performed at 3 years of age which showed established encephalomalacia in the frontoparietal area with mild prominence of the lateral ventricles, thin corpus callosum and loss of white matter volume. Follow-up MRI study was performed at 5 years of age. See Figure 16.

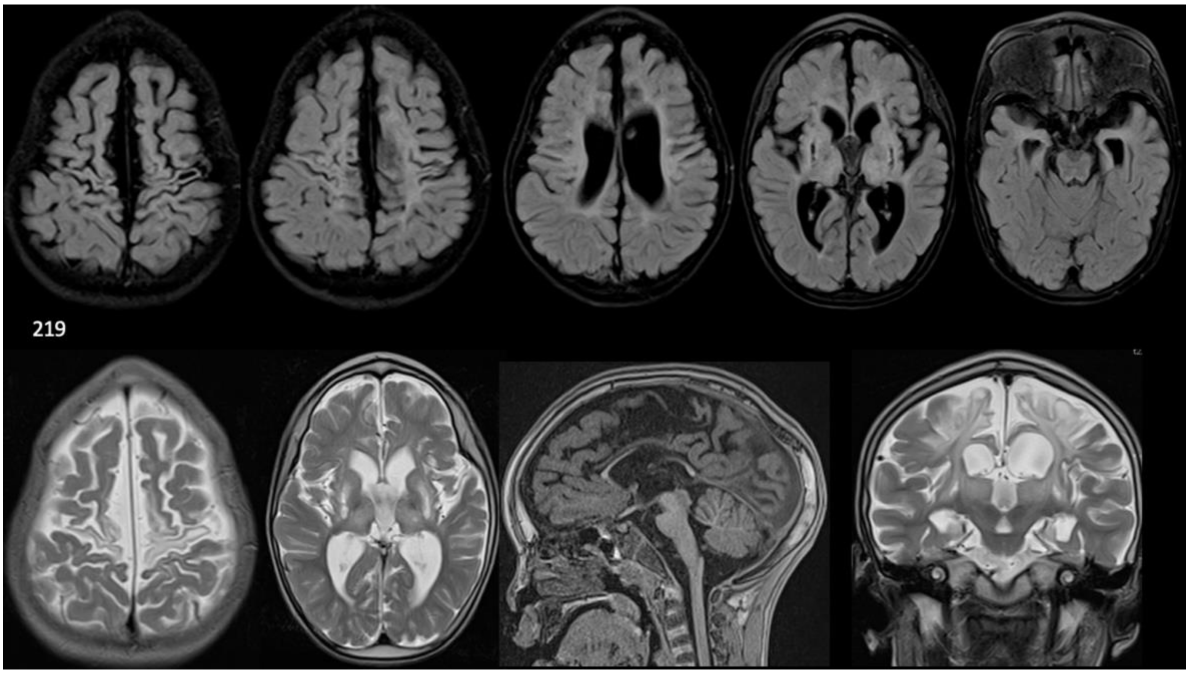

Figure 16 Collage of MRI images obtained for patient 219

Patient 229

A 30-year-old Para 1 Gravida 2 mum presented to a Level 2 local hospital in advanced labour. It was recorded that she was already 16 hours into labour when she presented. She had two visits to the clinic during her pregnancy and antenatal assessments did not reveal any abnormalities. She was known to be HIV positive on antiretroviral therapy. Her CD4 count was recorded above 500cells/mm^3. Prolongation of the first and second stage of labour was noted with decelerations identified by the midwife on the cardiotocograph. An early decision was made to proceed with caesarean section under epidural anaesthesia. A hypotonic female infant was extracted with low Apgar scores of 2/10 at 1 minute and 3/10 at 5 minutes. Oxygen was supplemented by nasal prongs. Head circumference measured 32cm and length was 46cm. There were early seizures noted. The blood glucose levels and other electrolytes were normal. Unfortunately, due to the local lack of expertise and imaging services, there was no early neonatal imaging available. MRI was performed at the age of 6 years. See Figure 17.

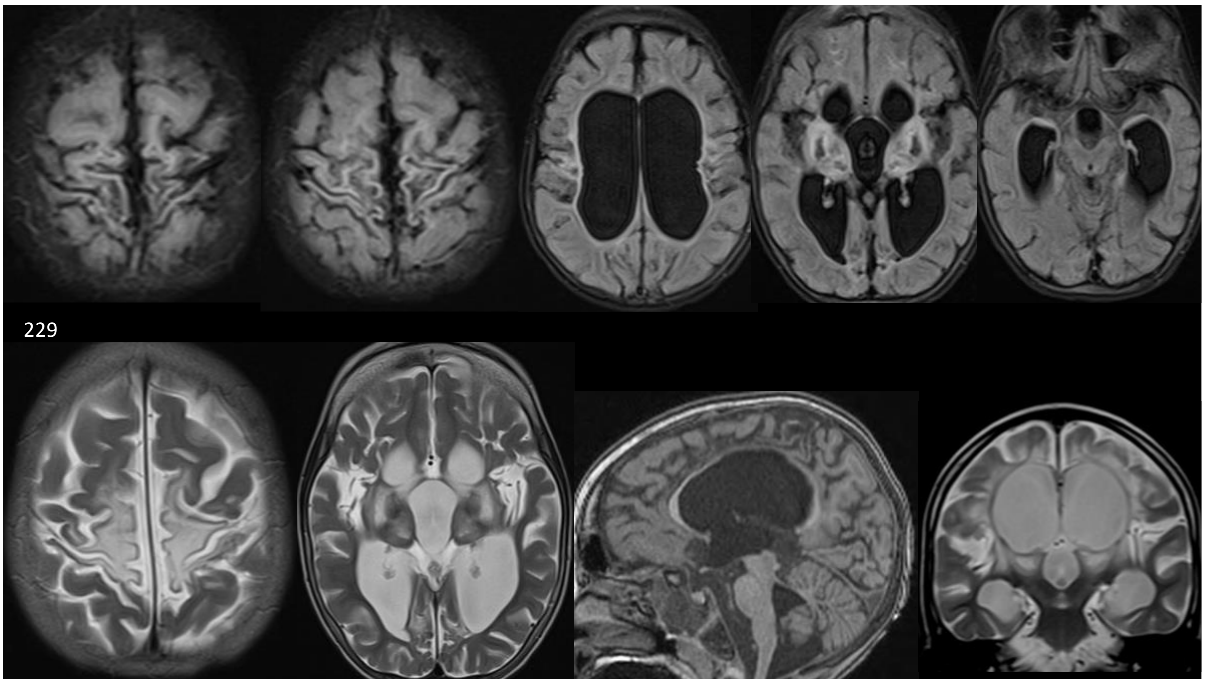

Figure 17 Collage of MRI images obtained for patient 229

Patient 234

The 38-year-old mother of this child, Para 1 Gravida 2, had an uneventful pregnancy with no antenatal medical complications documented during her clinic visits. A normal antenatal ultrasound was performed at 20 weeks gestation. She presented to the local Level 2 hospital at term in active labour. Severe prolongation of the second stage of labour was documented with attendant fetal distress. A caesarean section could not be performed as there was another operation underway in theatre and limited staff/ theatre facilities were noted. The exhausted mother finally delivered a flat female child by vaginal delivery augmented by an episiotomy. There was meconium staining of the liquor and meconium aspiration requiring suction and resuscitation. The Apgar scores at 1 and 5 minutes were calculated as 6/10 and 7/10 respectively. The hypotonic neonate required bag ventilation for several minutes before she continued to breathe spontaneously. Late on day 1 post-delivery, seizures were noted requiring phenobarbitone infusion. The blood glucose levels were recorded as normal. Baseline ultrasound on day 1 was normal. Repeat ultrasound on day 3 demonstrated cerebral swelling but no fluid collections or ventriculomegaly. A CT scan was performed at age of 5 months, and this revealed mild ventricular prominence related to central atrophy as well as thinning of the body of the corpus callosum. The MRI was performed at 3 years of age. See Figure 18.

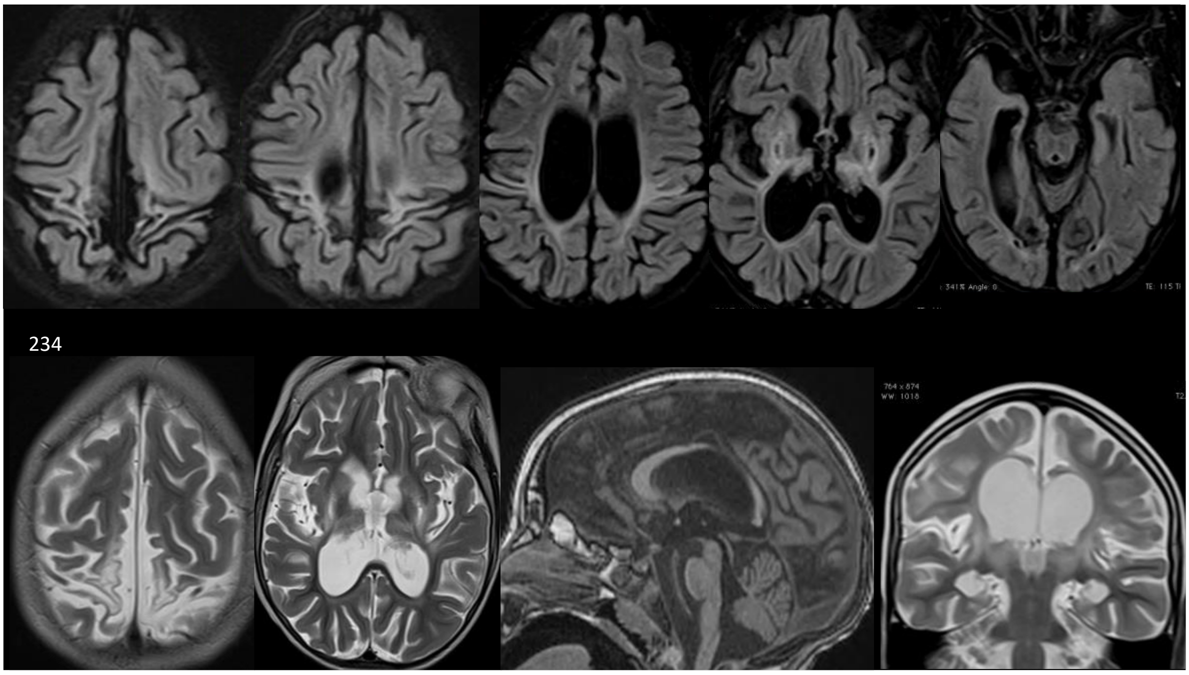

Figure 18 Collage of MRI images obtained for patient 234

Patient 297

A 25-year-old Para 0+1 Gravida 2 lady was well managed through her pregnancy by her treating obstetrician. She had had multiple visits to the obstetrician during her pregnancy considering her previous miscarriage. All her antenatal assessments, including a second trimester fetal ultrasound, did not reveal any significant abnormalities. She presented at 38 weeks-gestation to the private hospital, that she had booked to have her delivery performed at, in active labour. Prolongation of the second stage of labour was encountered when vaginal delivery was attempted and could not be augmented even by forceps delivery. Fetal distress, with early decelerations, was recorded on the cardiotocograph. The obstetrician decided to perform a caesarean section and an epidural was administered. A flat male child was extracted two hours after commencement of the second stage of labour. The birth weight was 3130g, the head circumference 36cm and the length 49cm. The Apgar scores were calculated as 1/10 at 1 minute and 3/10 at 5 minutes. The child required intubation and ventilation as well as anticonvulsant therapy for early and severe seizure activity. A cephalhematoma was attributed to the prolonged cephalo-pelvic disproportion or the forceps used. The child received head cooling. The blood glucose levels, and other electrolytes were normal. Serial ultrasound scans were performed; on day 1 showing no abnormalities and on day 3 cerebral oedema was reported. An MRI was performed at day 15 which showed perirolandic and basal ganglia changes reported due to acute profound ischemic injury. There were no features of metabolic disorders or neonatal leukodystrophy. A follow-up CT scan at 6 months showed no progressive changes. An MRI was performed at the age of 6 years. See Figure 19.

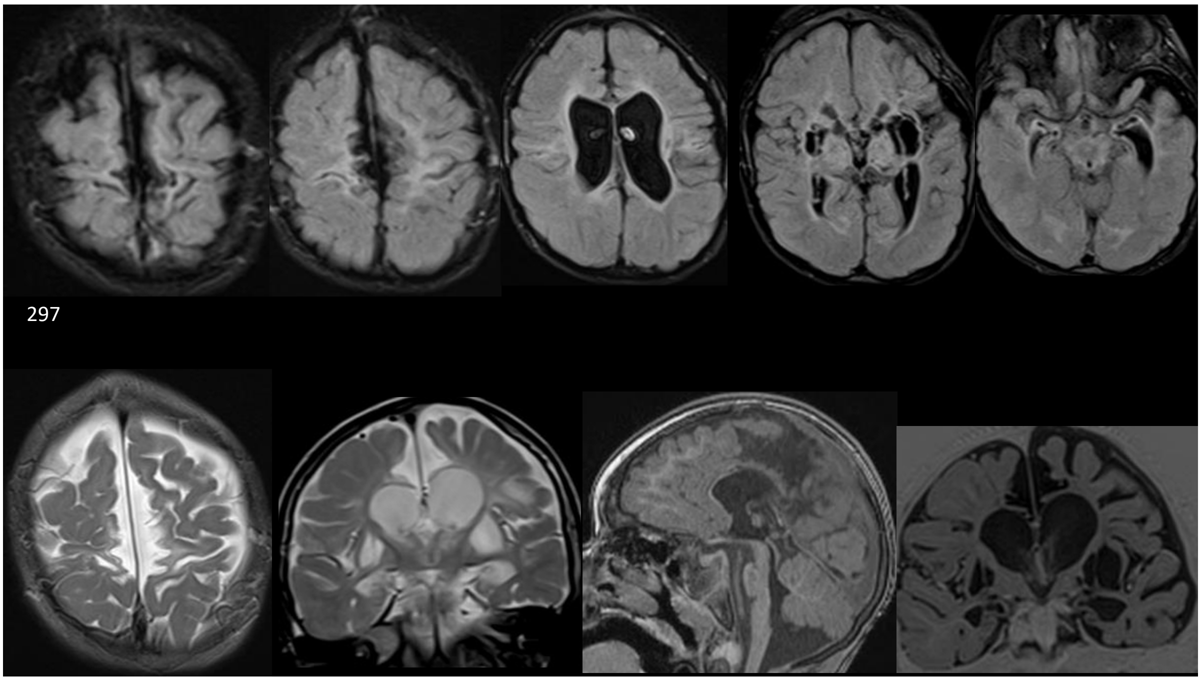

Figure 19 Collage of MRI images obtained for patient 297
